# Unlocking the in situ Li plating dynamics and evolution mediated by diverse metallic substrates in all-solid-state batteries

**DOI:** 10.1126/sciadv.add2000

**Published:** 2022-10-28

**Authors:** Can Cui, Hui Yang, Cheng Zeng, Siwei Gui, Jianing Liang, Ping Xiao, Shuhao Wang, Guxin Huang, Mingtao Hu, Tianyou Zhai, Huiqiao Li

**Affiliations:** ^1^ State Key Laboratory of Materials Processing and Die and Mould Technology, School of Materials Science and Engineering, Huazhong University of Science and Technology, Wuhan, Hubei 430074, P. R. China.; ^2^ Department of Mechanics, School of Aerospace Engineering, Huazhong University of Science and Technology, Wuhan, Hubei 430074, P. R. China.

## Abstract

The mechanisms of Li deposition behaviors, which overwhelmingly affect battery performances and safety, are far to be understood in solid-state batteries. Here, using in situ micro-nano electrochemical scanning electron microscopy (SEM) manipulation platform, dynamic Li plating behaviors on 10 metallic substrates have been tracked, and the underlying mechanisms for dendrite-free Li plating are elucidated. Distinct Li deposition behaviors on Cu, Ti, Ni, Bi, Cr, In, Ag, Au, Pd, and Al are revealed quantitatively in nucleation densities, growth rates, and anisotropic ratios. For Li alloyable metals, the dynamic Li alloying process before Li growth is visually captured. It is concluded that a good affinity for Li and appropriate lattice compatibility between the substrate and Li are needed to facilitate homogeneous Li plating. Our work not only uncovers the Li plating dynamics, shedding light on the design of solid-state batteries, but also provides a powerful integrated SEM platform for future in-depth investigation of solid-state batteries.

## Teaser

In situ Li plating dynamics on diverse metallic substrates in all-solid-state batteries are directly visualized and revealed.

## INTRODUCTION

Lithium metal battery (LMB) is regarded as the most promising high–energy density next-generation rechargeable battery because of the lowest negative potential (−3.040 V versus standard hydrogen electrode) and ultrahigh theoretical capacity of Li anode (*1*, *2*). However, the severe reactivity with liquid electrolyte and dendrite formation of Li anode bring great challenges to LMB’s safety and cycle life (*3*–*5*). Using solid-state electrolyte (SSE) instead of combustible organic liquid electrolyte is considered a fundamental way to improve the safety of LMBs (*6*–*11*).

Similar to lithium-ion batteries (LIBs), LMBs usually use LiCoO_2_, LiFePO_4_, LiNi*_x_*Co*_y_*Mn_1-*x*-*y*_O_2_, etc., as the cathodes, which are all at lithiated states initially (*12*–*15*). When assembling these cathodes into a cell of LMB, Li^+^ can be extracted from the cathode lattice during the first charging of the cell and deposited on the anode side even without any additional Li source, which can be acted as the anode material in the following cycles, enabling an anode-free solid-state battery (AFSSB). Such AFSSB avoids the direct use and operation of ultra-active Li metal; therefore, it can largely reduce the waste of Li metal and avoid harsh requirements of the environment for battery manufacturing (*16*–*18*). The anode-free design can maximize the volumetric and gravimetric energy density of the battery, for instance, the AFSSB developed by Lee *et al.* can boost the energy density over 900 Wh liter^−1^, which is much higher than that of LIB (751 Wh liter^−1^) (*19*, *20*). Therefore, AFSSB is regarded as the future direction of high–energy density solid-state batteries (*21*–*24*).

However, the performance of an AFSSB would greatly depend on the Li deposition behavior on the current collector at the anode side. Previous reports have demonstrated the vital influence of the substrate on the behavior of Li deposition in liquid electrolytes (*25*–*28*). For example, Cui’s group revealed a substrate-dependent Li growth phenomenon that manifested the highly selective deposition behaviors of Li on Cu and Au substrates in 1.0 M LiPF_6_ in ethylene carbonate (EC)/diethyl carbonate (*27*). The remarkable electrochemical influences of substrates on the Li deposition/plating potential and reversibility in 1.0 M LiPF_6_ in EC/ethyl methyl carbonate were also reported by Heligman and Manthiram (*28*). When the electrolyte of the battery changes from liquid to solid state, the circumstances for Li^+^ migration and Li deposition would radically change, so does the failure modes of the battery (*29*–*32*). However, most of the previous research are focused on liquid electrolyte system (*33*–*35*). The Li deposition behaviors and the underlying mechanisms in solid-state batteries with different substrates, which would overwhelmingly determine the performance of the AFSSB, are far to be understood, leaving an uncultivated but significant territory for us to explore.

Here, we set up an integrated in situ micro-nano scanning electron microscopy (SEM) manipulation platform, which can externally couple with an electrochemical testing system to successfully visualize the real-time Li deposition behaviors on different metallic substrates for all-solid-state batteries. It is found that, among 10 different substrate materials, Li deposits exhibit vertically dendrite-like Li growth on Cu, Ti, Ni, Cr, and Bi but present laterally particulate-like Li deposition on In, Ag, Au, Pd, and Al. The growth rates, distribution, orientation, anisotropic ratios (ARs), and nucleation densities of Li deposits on different substrates are quantitatively analyzed in detail. Moreover, we successfully track the different modes of alloying dynamics between Li and certain substrates before Li growth, which have never been intuitively captured in experiment before. The influence of the interaction between Li and substrate in solid-state batteries on the Li deposition behavior is discussed; in principle, an ideal substrate that satisfies both high affinity to Li and good crystallographic compatibility with Li could facilitate an in-plane, isotropic, and homogeneous Li growth. Our work not only provides real-time evidence of Li deposition behavior in solid-state battery system but also offers an integrated in situ SEM manipulation platform that can potentially make a breakthrough from qualitative to quantitative dynamic investigation and provide rational guidance for the future design of solid-state batteries.

## RESULTS

### In situ SEM manipulation platform for Li plating visualization

**Fig. 1.**
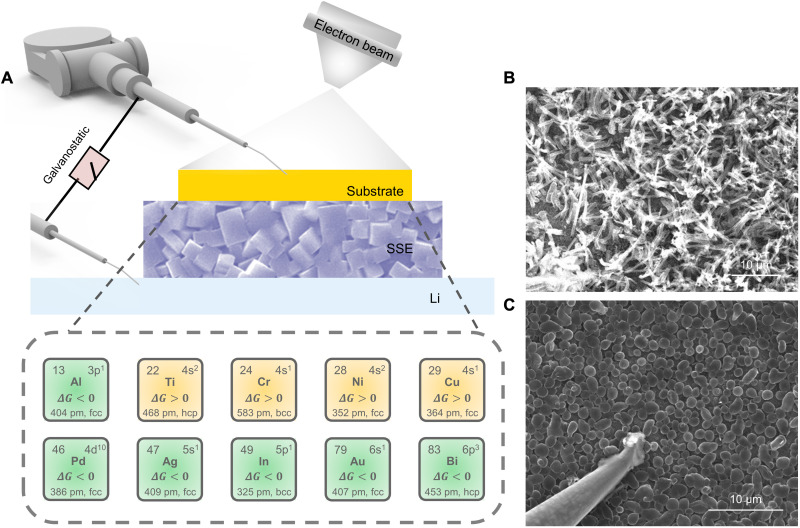
In situ SEM manipulation for Li plating visualization. (**A**) Schematic illustration of the in situ SEM manipulation platform. Ten different metallic substrates (Al, Ti, Cr, Ni, Cu, Pd, Ag, In, Au, and Bi) are listed as the research objects. (**B**) SEM image of the heterogeneous and dendrite-like growth mode, where Li is deposited on the Bi metallic substrate. (**C**) SEM image of the homogeneous and particulate-like growth mode, where Li is deposited on the In metallic substrate.

Figure 1A is the schematic illustration of the in situ SEM manipulation platform used to visualize Li plating. The micro-nano manipulators are set up in an SEM chamber and coupled with an external electrochemical workstation (fig. S1). The probes, which are planted in the front end of the manipulators, are fixed onto the two sides of the all-solid-state battery composed of a Li metal counter/reference electrode, a metallic substrate working electrode, and a Li_1.5_Al_0.5_Ge_1.5_P_3_O_12_ (LAGP) SSE. LAGP, with intimate contact with Li and relatively high Li^+^ conductivity, is chosen for the experiment (fig. S2). By electron beam evaporation, a circle-shaped metallic substrate microelectrode with a thickness of more than 100 nm is patterned onto LAGP SSE, which can serve as the substrate for Li deposition. Such configuration enables in situ observation of the dynamic behaviors of Li plating. We fabricate the metallic substrates with different thicknesses. It is found that as long as the evaporated metallic particles can form a continuous layer on LAGP SSE, the metallic substrates with different thicknesses exhibit similar deposition morphologies (figs. S3 and S4). For instance, on In substrates with 100, 200, and 250 nm thicknesses, Li deposits all exhibit uniform and particulate-like morphologies. Besides, with the increasing of the given current density, the nucleation density of Li deposits would increase (fig. S5). In addition, a current of 1 μA is applied in the following experiment for better in situ SEM observation.

Ten different metallic substrates (Al, Ti, Cr, Ni, Cu, Pd, Ag, Au, In, and Bi, as listed in Fig. 1A) are investigated, and the Li growth behaviors on these 10 substrates were systematically studied. The basic characterizations of 10 metallic substrates are given in figs. S6 to S8. A current of 1 μA is applied on the in situ device, and the discharging curves of the 10 metals with Li are given in fig. S9. Among all the metallic substrates, Ti, Cr, Ni, and Cu are proved to be lithiophobic metals, denoted by yellow squares, while Al, Pd, Ag, In, Au, and Bi are regarded as lithiophilic metals, indicated by green squares (*36*). Our experiment results demonstrate different Li deposition behaviors on these substrates. The Li growth modes can be generally classified into two distinctive categories: one is the heterogeneous dendrite-like growth mode (Fig. 1B and movie S1), and the other is the homogeneous and uniform particulate-like growth mode (Fig. 1C and movie S2). The dynamic process of Li nucleation and growth on the 10 metallic substrates are given in figs. S10 and S11. For Cu, Ti, Ni, and Bi substrates, fast rambling dendrite-like Li growth can be observed, while metallic Cr shows only local dendrite formation accompanied with cracking failure of the SSE, consistent with the obvious fluctuation in the corresponding discharging curves in fig. S9. In sharp contrast, homogeneous Li nucleation and particulate-like Li growth with uniform size are observed on In, Ag, Au, Pd, and Al metallic substrates, with no formation of Li dendrite. Because our in situ research adopts the same battery configuration as the practical one, the acquired results are therefore believed to be reliable and comparable with the actual situation and circumstances of a real solid-state battery.

### Dynamic Li deposition behaviors on different substrates

**Fig. 2.**
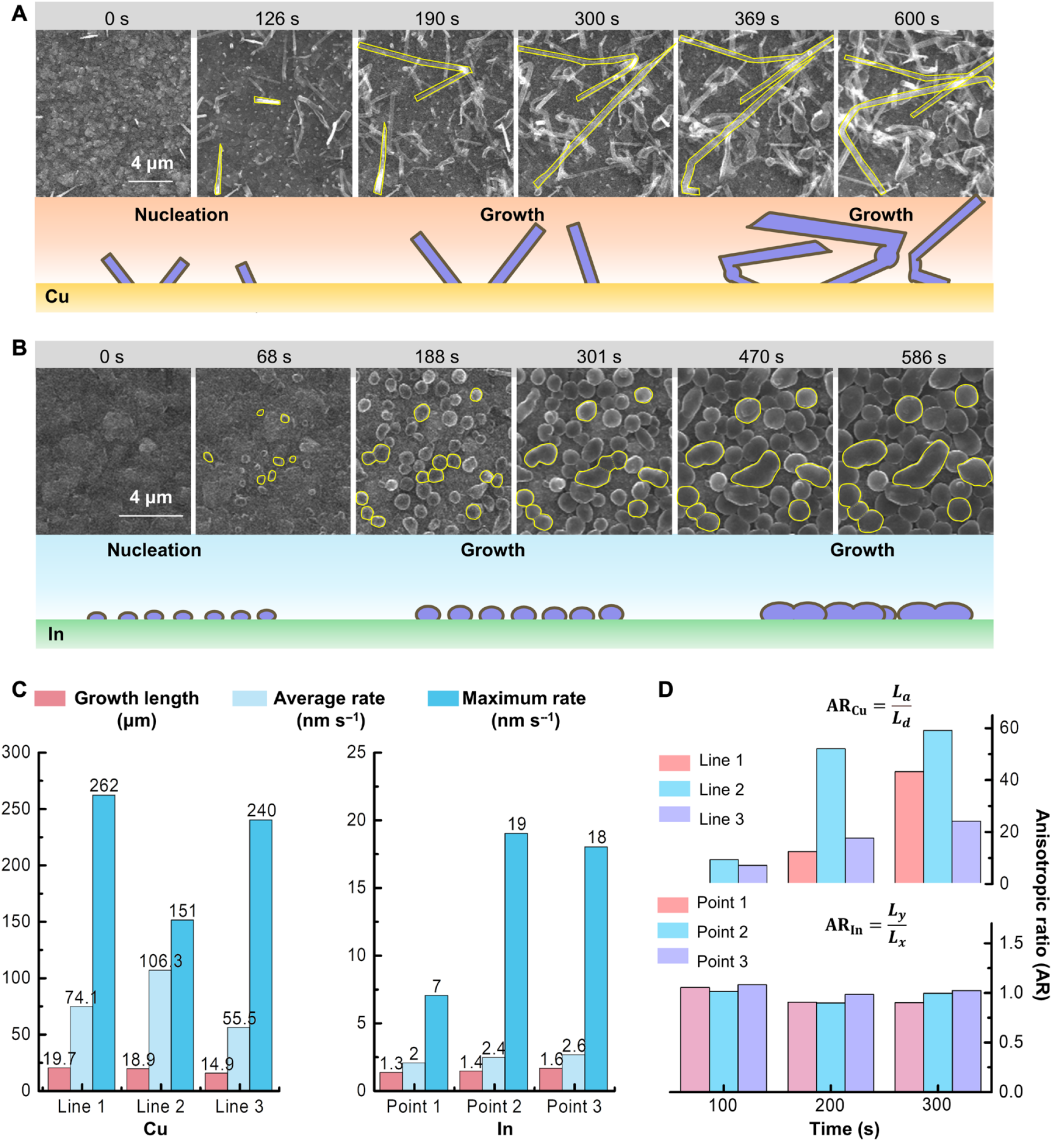
Dynamic Li deposition behaviors on different substrates. (**A**) Time-series SEM images and corresponding schematic illustration of Li deposition behavior on the Cu substrate. (**B**) Time-series SEM images and schematic illustration of Li deposition behavior on the In substrate. (**C**) Comparison of Li growth lengths, average growth rates, and maximum instantaneous growth rates of the three randomly chosen Li deposits on Cu or In substrates. (**D**) Anisotropic ratios of the chosen Li deposits on Cu and In substrates at the plating time of 100, 200, and 300 s.

For a better understanding of the dynamic Li deposition behavior, we selected two representative metals, i.e., Cu and In, for the case study of the dendrite-like and particulate-like Li growth modes, respectively. By applying galvanostatic current onto the microprobes through an external electrochemical workstation for 10 min, in situ observation can be made for the morphology evolution of Li plating. Figure 2A shows the time-series SEM images and the corresponding schematic illustration of Li deposition behavior on the Cu substrate. It can be clearly observed that Li whiskers first nucleate randomly on the substrate. With the proceeding of deposition, the short rod-like Li whiskers tend to grow into long needle-like dendrites via a root growth mode (*37*). Meanwhile, the later-formed whiskers will squeeze out from the substrate and push forward the former parts. Such Li deposition on the substrate shows a typical out-of-plane dendrite-like growth mode. During the continuous elongation of Li whiskers, the transformation of grain growth orientation can lead to the kinking of whiskers, namely, excessive deformation of the whiskers. In contrast, the growth of Li on the In substrate exhibits a particulate-like morphology, as revealed by the time-series SEM images and the schematic illustration in Fig. 2B. The similar-sized Li particles nucleate uniformly on the In substrate. As the deposition moves forward, the diameters of the Li particles increase, along with the particulate shape well kept all the time, suggesting a uniform growth rate in all directions on the substrate. When adjacent particles come into contact with each other, the deposited Li particles gradually merge together and cover the whole surface of the substrate. Obviously, the Li deposition on the In substrate reveals an in-plane particulate-like growth mode, quite different from the out-of-plane dendrite-like growth mode on the Cu substrate.

Taking advantage of the in situ electrochemical SEM micro-nano manipulation platform, we also study the growth rates of Li deposits on Cu and In substrates by tracking the evolution of Li growth direction and length. As shown in fig. S12 (A to D), three whiskers located at different positions are selected for the measurement of growth length of Li on the Cu substrate, while for the case with the In substrate, three particles are chosen (fig. S13A). The evolution of the measured growth length (*L*) of Li deposits on Cu and In substrates is given in figs. S12E and S13B, respectively. Further, the growth rate of Li deposits is calculated by Eq. 1 and given in figs. S12F and S13C

$$v=\frac{\mathrm{dL}}{\mathrm{dt}}$$ (1)

The growth length, average growth rate, and maximum growth rate of each individual Li deposit on the Cu or In substrate are compared in Fig. 2C. For the three randomly chosen Li whiskers on Cu, the growth lengths are measured to be 19.7, 18.9, and 14.9 μm, resulting in an average growth length of 17.8 μm. The derived average growth rates of each Li whisker differ markedly, ranging from 55.5 to 106.3 nm s^−1^, and the maximum instantaneous growth rate of Li whiskers on the Cu substrate is calculated up to 262 nm s^−1^ when going through the transformation of the growth orientations (*38*). Such an extremely fast and heterogeneous Li deposition would easily trigger short circuit in batteries. In sharp contrast, the growth lengths of the particulate-like Li deposits on the In substrate are more uniform and homogeneous, with an average growth length of 1.4 μm. The Li deposits deliver an average growth rate of 2 to 3 nm s^−1^, and the maximum instantaneous growth rate of Li particle is measured to be 19 nm s^−1^ at the beginning of the nucleation stage, much lower than that for out-of-plane dendrite-like growth mode, indicating a much steadier in-plane particulate-like growth mode on the In substrate. Apart from the huge differences in growth rates, the growth anisotropies of Li deposits on Cu and In are investigated. As exhibited in Fig. 2D, the ARs of the chosen Li deposits on Cu and In substrates at the plating time of 100, 200, and 300 s are calculated according to Eqs. 2 and 3

$${\text{AR}}_{\text{Cu}}=\frac{L_{a}}{L_{d}}$$ (2)

$${\text{AR}}_{\text{In}}=\frac{L_{y}}{L_{x}}$$ (3)

where *L_a_* and *L_d_* denote the growth lengths along the axial direction and the radial direction of a Li whisker, respectively; *L_x_* and *L_y_* are measured along the in-plane two perpendicular diametric directions of a Li particle. It can be clearly seen that Li on the Cu substrate tends to grow in the axial direction. The AR can be calculated up to 59.0 at the plating time of 300 s. The large divergences among different whiskers are quite obvious, demonstrating the preferred growth orientation and extreme inhomogeneity of Li growth on the Cu substrate. However, the ARs of the randomly selected three Li deposits on the In substrate are always close to 1.0 (± 0.2) and show no apparent time-dependent evolution, clearly revealing the steady and isotropic growth mode of the Li particles on the In substrate. Such quantitative information on Li growth lengths, rates, and ARs on different substrates can provide new insights for understanding the electrochemical kinetics in solid-state batteries.

**Fig. 3.**
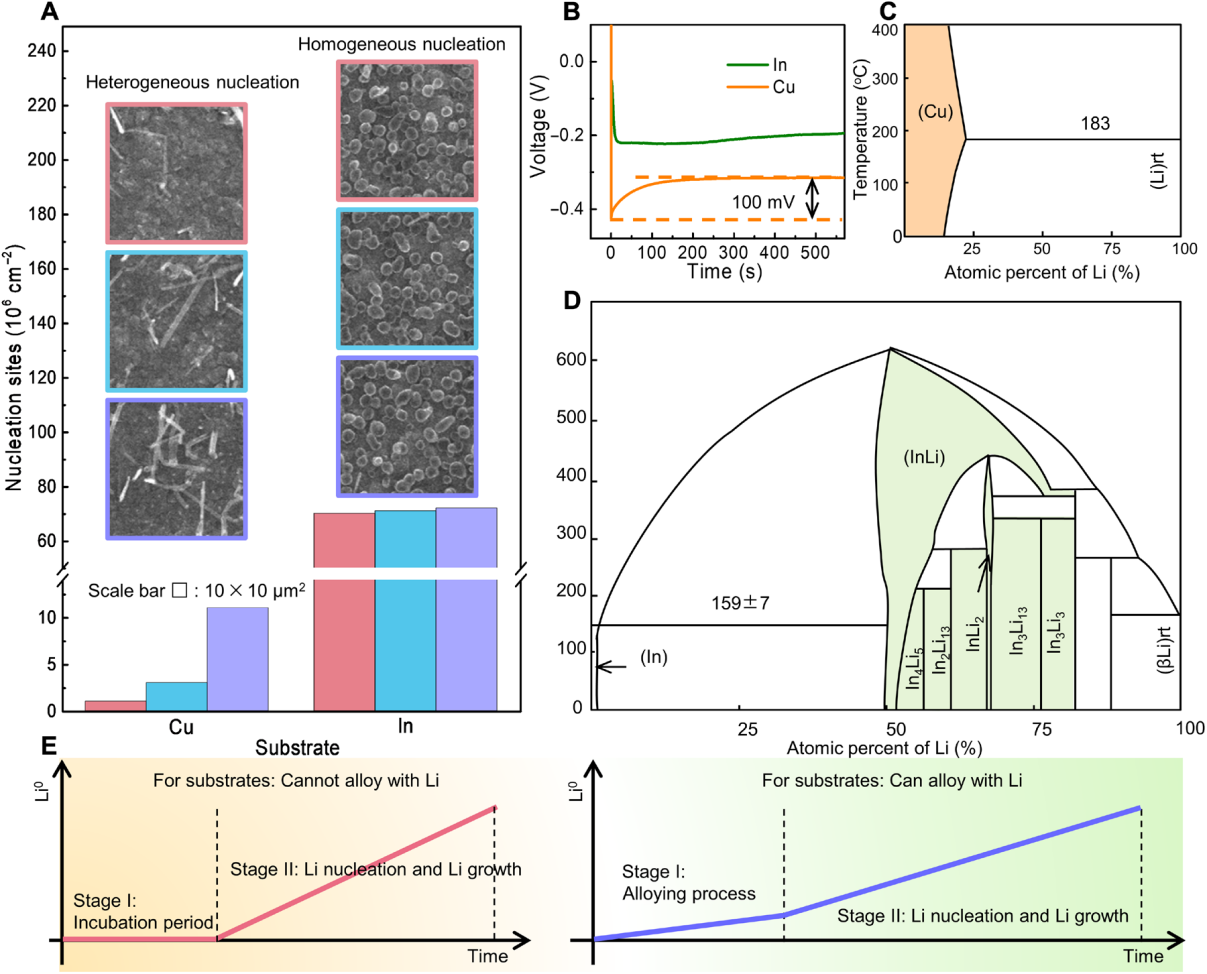
Li nucleation on different substrates. (**A**) The densities of Li nuclei on the Cu and In substrate and the insets are the randomly selected three 10 μm × 10 μm square frames on each substrate. (**B**) Voltage profiles of Li deposition on Cu and In substrates. (**C**) Binary alloy phase diagram of Li-Cu. (**D**) Binary alloy phase diagram of Li-In. (**E**) Schematic diagram of possible deposition processes for both Li nonalloyable and alloyable metallic substrates.

Further, the Li nucleation kinetics on different substrates are investigated. Since the distribution of Li nuclei is a vital evaluation criterion for uniform Li deposition, the densities of Li nuclei on Cu and In substrates are compared. As shown in Fig. 3A, by randomly selecting three 10 μm × 10 μm square frames on each substrate, the number of Li nuclei within a square frame is statistically counted. It can be observed that Li plated on the Cu substrate shows a density of nuclei no more than 1.1 × 10^7^ cm^−2^, and at some places, it is even as low as 1.0 × 10^5^ cm^−2^, demonstrating the extremely uneven Li deposition on the Cu substrate. For the case with the In substrate, Li nuclei show a much uniform distribution with an average density of 7.0 × 10^7^ cm^−2^, which is nearly seven times higher than that on the Cu substrate. In addition, as revealed by the voltage profiles of Li deposition on the two metallic substrates (Fig. 3B), there is an apparent voltage dip at the initial stage of Li deposition on the Cu substrate and then followed by a flat voltage plateau. The gap between the voltage dip and plateau is defined as the overpotential for Li nucleation, which is measured to be 100 mV, suggesting a huge energy barrier for Li nucleation on the Cu substrate, which may well explain the slow and sluggish nucleation of Li dendrite on the Cu substrate. In contrast, the voltage profile of Li on the In substrate shows a flat voltage turning with no initial voltage spike, illustrating a much smaller overpotential and thus a negligible energy barrier for Li nucleation.

The distinct differences in Li deposition behaviors between Cu and In are thought to be related to their different affinities to Li. As shown in Fig. 3C, Li shows no solubility in the Cu substrate thermodynamically (*39*). Thus, the Cu substrate cannot alloy with Li, suggesting the lithiophobic characteristic of Cu. This may help to explain the rather low nucleation density on the Cu substrate and heterogeneous out-of-plane dendrite-like Li growth mode. In contrast, Li could react with the In substrate and form various Li-In alloy phases, meaning a wide range of solubility in In, as illustrated in Fig. 3D. When Li^+^ is reduced to Li atom, it would first dissolve into the In substrate to form Li-In alloy, which endows the substrate with high lithiophilicity to facilitate the Li plating, resulting in high Li nucleation density with nearly zero overpotential as well as in-plane Li growth paralleled to the surface of the In substrate.

On the basis of the above observations, the possible deposition processes for both Li nonalloyable and alloyable metallic substrates are deduced in Fig. 3E. For the Li nonalloyable metallic substrates in which Li shows no solubility, the Li deposition process on them usually consists of two stages: stage I, a waiting period for nucleation incubation with energy cumulation, and stage II, Li nucleation and growth after overcoming the energy barrier. For the substrates that are alloyable with Li, they will first experience the spontaneously alloying step at the beginning (stage I) and then followed with the Li nucleation and growth process (stage II).

### Dynamic Li alloying process on different substrates

**Fig. 4.**
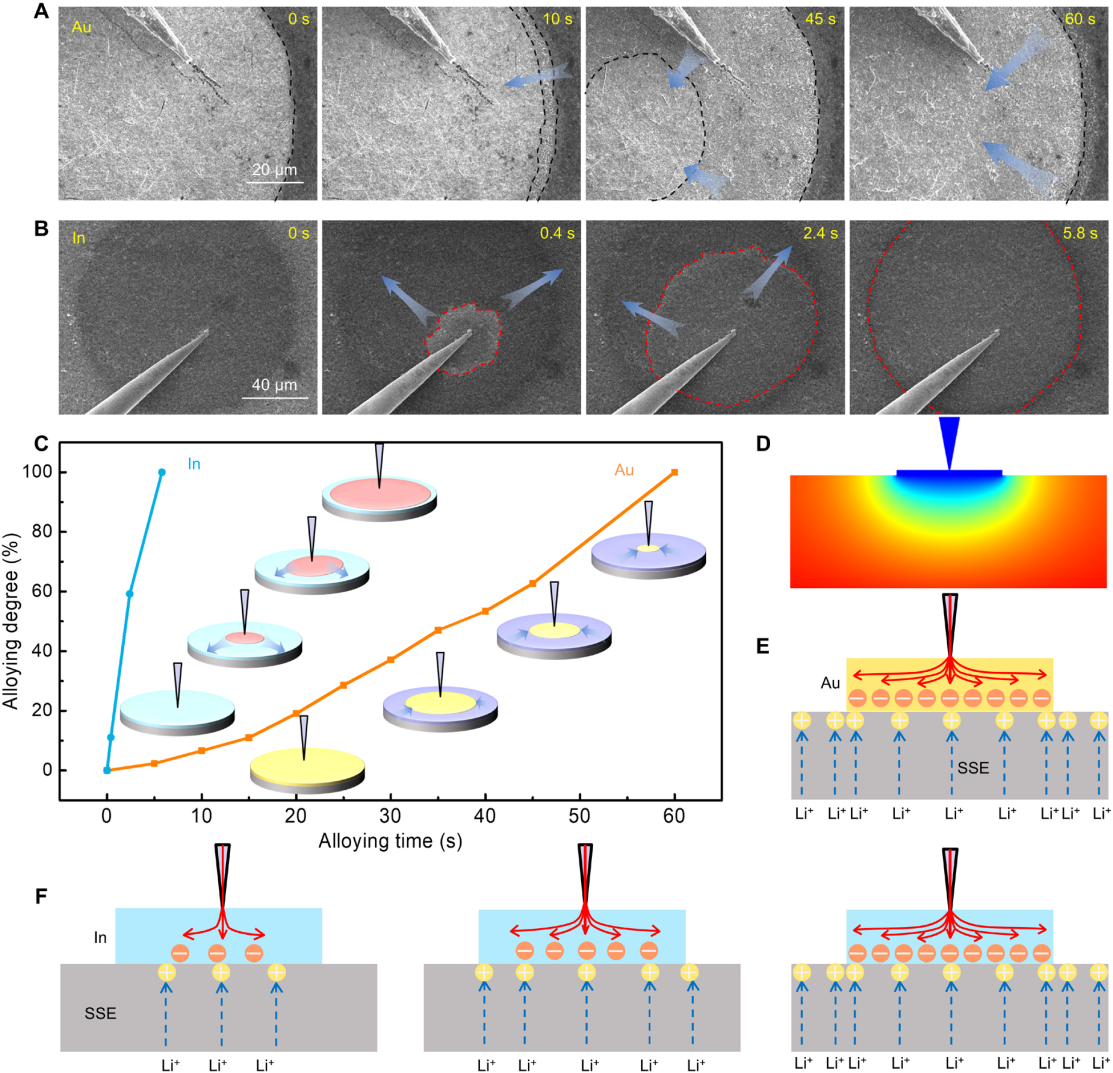
Dynamic Li alloying process on different Li alloyable metallic substrates. (**A**) Time-series SEM images of Li-Au alloying phenomenon. (**B**) Time-series SEM images of Li-In alloying phenomenon. (**C**) Li-Au and Li-In alloying degree versus time, and the insets are the schematic illustrations of Li-Au and Li-In alloying. (**D**) Potential distribution of the SSE beneath the Au substrate (cross-sectional view). (**E**) Schematic diagram of Li^+^ and electron distribution at the Au||SSE interface. (**F**) Schematic diagrams of Li^+^ flux and electron distribution at the In||SSE interface.

The dynamic Li alloying process on different Li alloyable substrates, i.e., Au and In, is also studied via in situ movie by recording the initial discharging process before Li nucleation on the substrates. As shown in movies S3 and S4, the alloy formation process can be tracked by the fast and dynamic contrast variations of the substrate microelectrode at the very beginning of discharging. The imaging principle of SEM relies on the secondary electron emission process, where many free electrons will be generated when high-energy incident electron beam is injected into the sample, 90% of which come from the outer valence electrons of the sample’s atom. In addition, the contrast of SEM images is related to the yield of secondary electrons. During the alloying process before Li nucleation and growth, electrons are conducted from the microprobe to the metallic substrate, while Li ions diffuse from LAGP SSE to the surface of the substrate. Once the Li ions obtain electrons at the interface of the substrate and LAGP, the generated Li atoms dissolve into the metallic substrate to form alloys. With the progress of the lithiated process, the surface conditions of the sample are doomed to change. Thus, we can obviously observe the changes in contrast variations from movies S3 and S4. Figure S14 tags the lithiated area and nonlithiated area for clarity. The alloying phenomena of Li with Au and In are quite distinct from each other. Taking the Au substrate for example (Fig. 4A and movie S3), the alloying reaction starts from the edge of the microelectrode and gradually moves forward to the center. However, for the In substrate in Fig. 4B and movie S4, the alloying reaction begins at the tip of the probe and then spreads out toward the edge of the microelectrode. As illustrated in Fig. 4C, the whole Li-Au alloying reaction lasts for 60 s, while the Li-In alloying process is finished within 6 s. Using the following Eq. 4

$$v=\frac{\mathrm{dS}}{\mathrm{dt}}$$ (4)

where *S* is the alloyed surface area of the microelectrode, the average alloying rate of In can be estimated to be 2.57 × 10^3^ μm^2^ s^−1^, which is one order of magnitude higher than that of Au (2.57 × 10^2^ μm^2^ s^−1^).

To better understand the alloying process, finite element simulation is conducted to study the potential and electrical field distributions in SSE with Au electrode in fig. S15. As Au has an excellent conductivity, the electrons from the microprobe could spread out fast enough and cover the whole substrate to form a uniform distribution. In such case, the potential field exhibits a bowl-like distribution in the SSE beneath the Au substrate, as seen from the cross-sectional view of the simulated model in Fig. 4D and fig. S15B. In addition, the distribution of electrical field in SSE (fig. S15C) reveals an apparent edge effect for Au||SSE. The electrical field intensity near the edge of the substrate is much higher than that in the center region. Therefore, the Li^+^ flux distribution would be more intensive near the edge and sparse beneath the center of the substrate (Fig. 4E), leading to the Li alloying process propagating from the edge to the center of the Au substrate.

In contrast, the relatively slow electron transportation restricts the Li alloying process in the In substrate. As shown in fig. S16, based on the conductivity measurements by four-point probe method, the In substrate shows a high resistivity of 2.36 × 10^7^ ohms, much higher than that for the Au substrate (1.67 ohms). Such high resistivity of the In substrate makes the injected electrons hardly spread out over the whole substrate within a short time; thus, the electrons tend to congregate at the tip of the microprobe, leading to the Li alloying reaction starting first at the probe tip contact point. With the spreading of electrons, the Li alloying reaction front can sweep from the probe tip contact point toward the edge of the substrate, as exhibited in Fig. 4F.

It is the later one between ion migration and electron transportation that determines the Li alloying patterns in different metallic substrates. For the Au substrate, the Li-Au alloying process is mainly affected by ionic migration, while the electron transportation plays a critical role in the alloying process of the In substrate. In terms of the alloying rates, it shows a much higher reaction rate on the In substrate than on the Au substrate, which might relate to the different work functions of these two substrates. As given in fig. S16D, the work function of In is 4.12 eV, lower than that of Au (5.1 eV). Thus, it is easier for Li^+^ to obtain electrons from the In substrate than the Au substrate, as a larger value of work function usually suggests a harder escape of electrons from the metal. This explains why Li alloying reaction with the In substrate spends much less time than the Au substrate. Besides, in situ x-ray diffraction (XRD) technique is also used to investigate the Li-Au and Li-In alloying process, as exhibited in figs. S17 and S18. Au experiences the intermetallic phases of LiAu_3_ and Li_3_Au during the alloying process. In addition, for In, the intermetallic phases of LiIn, Li_2_In, Li_3_In_2_, and Li_13_In_3_ occur during the alloying stage. Owing to the in situ SEM configuration coupling with an electrochemical testing system, our work provides direct visual proof of the initial Li alloying step on metallic substrates, and more intensively, we uncover the dynamic differences of such alloying process between different substrates in alloying rates and controlling mechanisms.

### Relationship between Li deposition behavior and diverse substrates

**Fig. 5.**
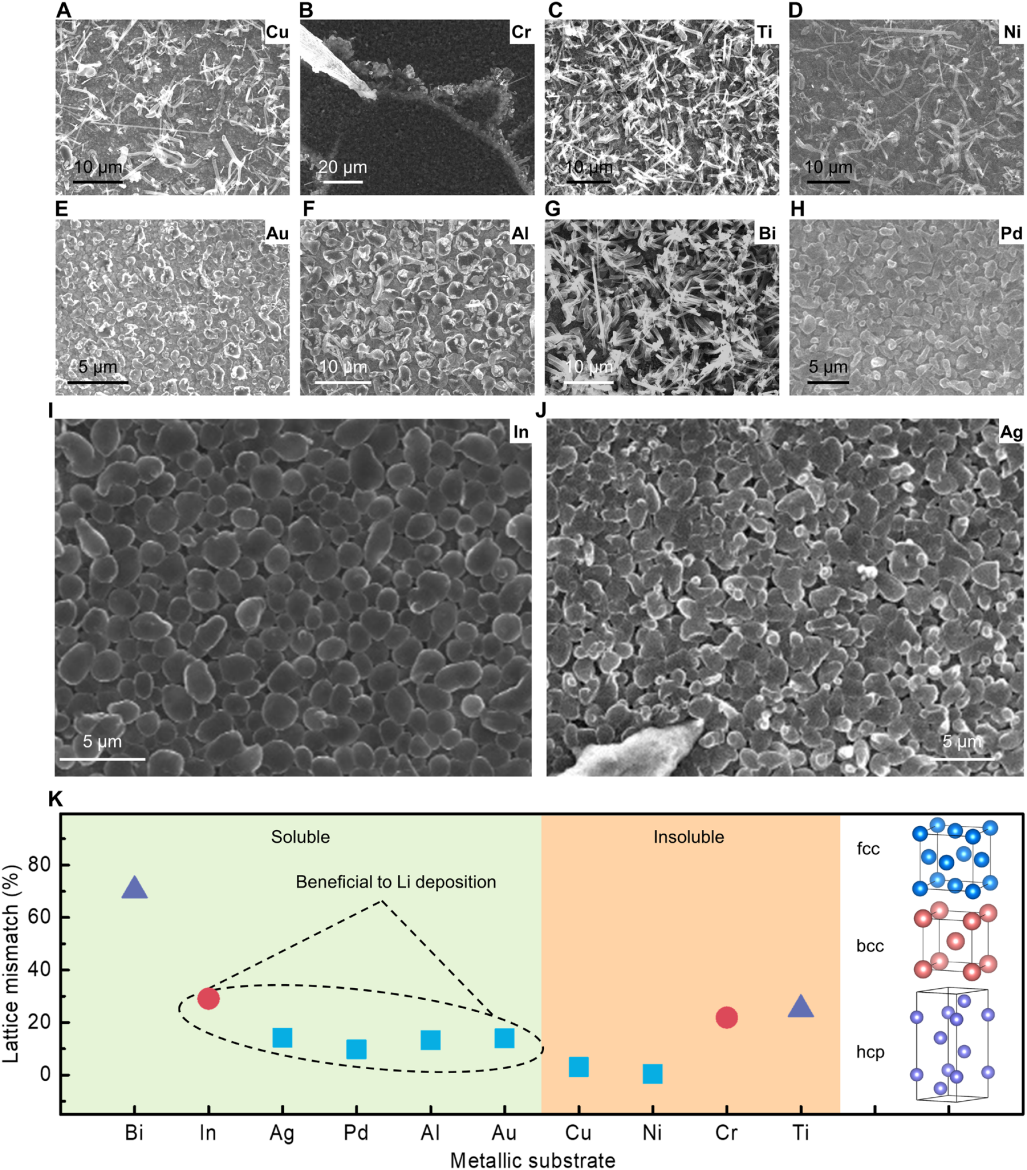
Relationship between Li deposition behavior and diverse substrates. (**A** to **J**) Deposited Li morphologies on (A) Cu, (B) Cr, (C) Ti, (D) Ni, (E) Au, (F) Al, (G) Bi, (H) Pd, (I) In, and (J) Ag. (**K**) Lattice mismatch between Li crystal and different metallic substrates.

Moreover, we summarize the Li growth behaviors on the 10 kinds of metallic substrates used in our in situ SEM investigation, including Cu, Cr, Ti, Ni, Au, Al, Bi, Pd, In, and Ag. As illustrated in Fig. 5 (A to J), the Li deposition mainly exhibits two morphologies, namely, the out-of-plane dendrite-like growth and the in-plane particulate-like growth. According to the binary diagrams of these 10 metals with Li (*39*), metallic Cu, Cr, Ti, and Ni are insoluble for Li metals, while Au, Al, Bi, Pd, In, and Ag metallic substrates are soluble for Li metal, which suggests that the solid diffusion of Li in the former four metals might be more difficult than that in the latter six metals. As shown in Fig. 5 (A to D) for Cu, Cr, Ti, and Ni that are nonalloyable with Li, it is found that all the four metallic substrates cannot realize homogeneous Li deposition. Instead of the large-area and long dendrite-like growth, Li dendrites form merely around the tip of microprobe on the Cr substrate, and further extra Li deposition would lead to cracking failure of the SSE (*40*). Among the metals that can alloy with Li, uniform and particulate-like Li deposition is observed on Au, Al, Pd, In, and Ag metallic substrates, showing that these metals have a superior affinity to Li. It should be noted that Bi, among the alloyable metallic substrates, demonstrates exceptional Li deposition behaviors. Although it can form Li-Bi alloy with Li, it also behaves in dendrite-like Li growth. Such exceptions revealed by Bi indicate that, besides Li solubility, there should be other factors affecting the Li growth behavior.

Considering that the plating of Li also undergoes a solid nucleation and growth process, the crystallographic compatibility between Li and its growth substrate might play an important role. For the 10 metals, their crystal structures can be categorized into three groups (Fig. 5K): face-centered cubic (fcc; denoted as blue squares), body-centered cubic (bcc; denoted as red circles), and hexagonal close-packed (hcp; denoted as purple triangles), respectively. The fundamental physical properties and key parameters of the metals are listed in table S1. The lattice mismatch between Li crystal and different substrate metals is calculated by Eq. 5

$$\delta =\frac{|a_{M}-a_{\mathrm{Li}}|}{a_{M}}$$ (5)

where *a_Li_* and *a_M_* are the lattice constant of Li and metallic substrate materials, respectively. For hcp metals, the calculation of lattice mismatch applies the long axis (axis *c*). As shown in Fig. 5K, Bi exhibits a large lattice mismatch of 70.3%. For the other nine metals, their lattice mismatches are all within 30%. Although Bi can form Li-Bi alloy, the huge lattice mismatch still brings a high energy barrier for the in-plane growth of Li nuclei along the Bi substrate; thus, the deposited Li prefers growing vertically into dendrites (Fig. 5G). On the basis of these observations, it is suggested that an ideal substrate for homogeneous Li deposition should simultaneously have a certain solubility and good lattice compatibility with Li; on the one hand, the formation of Li-Metal alloy enables the substrates to have more affinity for Li, thus lowering the overpotential for Li nucleation and providing uniform and abundant Li deposition sites for Li plating. Good lattice compatibility and high affinity for Li could conduce to an in-plane and isotropic Li growth rather than out-of-plane and anisotropic Li growth.

## DISCUSSION

In summary, using in situ micro-nano SEM manipulation platform coupled with an electrochemical workstation, we systematically investigate the Li deposition behaviors on 10 kinds of metallic substrates with LAGP as the SSE in all-solid-state batteries. The results reveal that Li growth on Cu, Ti, Ni, Bi, and Cr exhibits typical out-of-plane dendrite-like morphology, while it shows in-plane particulate-like pattern on In, Ag, Au, Pd, and Al. A detailed study finds that the Li growth rate on the Cu substrate can be up to 262 nm s^−1^, nearly 14 times faster than that on the In substrate (19 nm s^−1^). However, in contrast to the isotropic growth rates (AR = 1.0 ± 0.2) of Li on the In substrate, the highly anisotropic growth mode (AR_max_ = 59) is observed for Li on the Cu substrate. Moreover, before Li nucleation and growth, Li alloyable metals (Au and In) demonstrate distinct Li alloying dynamics in both alloying reaction rates and alloying directions, which are attributed to their different work functions and rate-controlling mechanisms of charge carriers. On the basis of the Li deposition behaviors on 10 metallic substrates, it is concluded that the affinity for Li and good lattice compatibility with Li play important roles in homogeneous and in-plane Li plating, facilitating uniform and lateral Li deposition. Our work not only uncovers the Li alloying and deposition dynamics with quantified details that have never been intuitively captured before, shedding light on the design of solid-state battery systems, but also provides a general powerful integrated micro-nano SEM manipulation platform for future in situ investigation of solid-state batteries.

## MATERIALS AND METHODS

### Material fabrication

The LAGP (purchased from Hefei Kejing Material Technology Co. Ltd.) powders were pressed into pellets and then sintered in furnace at 900°C for 3 hours. The thickness of the LAGP pellet used in experiment is 1.0 (± 0.1) mm.

### Fabrication of metallic microelectrodes

The circle-shaped microelectrodes (Cu, Ti, Ni, Bi, Cr, In, Ag, Au, Pd, and Al) in a diameter of 140 μm are patterned and evaporated on LAGP pellets by electron beam evaporation (Nexdep, Angstrom Engineering) with the help of a custom-tailored mask. The thicknesses of the metallic microelectrodes used in the experiments are from 50 nm to 2 μm. In addition, the chamber pressure during evaporation is required to be below 5 × 10^−8^ mbar.

### Fabrication of in situ device

The in situ device is fabricated with a sandwiched Li-LAGP-metal structure. The metallic substrates are evaporated on the LAGP surface. In addition, Li foil is pressed on the other side of LAGP SSE and kept for 3 to 5 min to guarantee intimate contact between Li and LAGP SSE. The whole fabrication process is completed in an argon-filled glovebox. The in situ device is transferred into SEM chamber by a well-sealed transfer box to prevent the influence of air and water.

### Characterizations

The morphology observation was characterized with a field-emission scanning electron microscope (FESEM) (Quanta 650 FEG). FESEM coupled with manipulators (MM3A-EM, Kleindiek Nanotechnik) and electrochemical workstation (CHI660E) built the in situ micro-nano manipulation SEM system with electrochemical analysis tests. The phase structures and metallic substrates are characterized by XRD [Bruker AXS D2 Advance with Cu Kα radiation (λ = 1.54178 Å)], x-ray photoelectron spectroscopy (AXIS-ULTRA DLD-600W), and transmission electron microscopy (FEI Tecnai JEOL 2100).

### In situ SEM setup

A microelectrode module with two micro-nano manipulators was fixed inside the SEM chamber. The probes made of tungsten were assembled into the front end of the manipulators. The manipulators can move precisely from microscale to nanoscale in *x*, *y*, and *z* orientations driven by the control unit of the piezoelectric ceramics. The manipulation wires were connected with the flange outside of the SEM chamber and then connected to the electrochemical workstation (CHI660E). Thus, the in situ micro-nano manipulation SEM with electrochemical testing system was constructed.

### Electrochemical measurements

The galvanostatic Li deposition was carried out with the current of 1 μA for 10 min by an electrochemical workstation (CHI660E). The electrochemical impedance spectroscopy measurement is conducted by Solartron Electrochemical Interface SI 1260 with a frequency of 10 MHz to 0.1 Hz. The cells were all tested at room temperature.

### Data analysis and processing

The growth length of Li deposits (*L*) and the alloyed surface area (*S*) can be measured and extracted from time-series SEM images by ImageJ software. In addition, the growth rate is the first-order derivative of growth length versus time; similarly, the alloying rate is the first-order derivative of alloyed surface area versus time, as illustrated in Eqs. 1 and 4, respectively

$$v=\frac{\mathrm{dL}}{\mathrm{dt}}$$ (1)

$$v=\frac{\mathrm{dS}}{\mathrm{dt}}$$ (4)

### Finite element simulation

Using COMSOL Multiphysics software, the finite element simulation was conducted to investigate the potential and electrical field distributions in the Au||SSE||Li system. According to our experimental setup, the following electrostatic equations are solved with pertinent material properties and boundary conditions

E=−∇V (6)

$$\nabla \cdot J=Q_{j,V}$$ (7)

$$J=\sigma E+J_{e}$$ (8)

where *V* and **E** are the electric potential and electric field intensity, respectively; **J** is the electric current density, with **J***_e_* as an externally generated current density; σ denotes the electrical conductivity; and *Q*_*j*,*V*_ represents the current sources.
