# Supplementary material for: Unlocking the in situ Li plating dynamics and evolution mediated by diverse metallic substrates in all-solid-state batteries
Source: Sci Adv. 2022 Oct 28;8(43):eadd2000. doi: 10.1126/sciadv.add2000 (PMC9616501; doi:10.1126/sciadv.add2000)
Supplement: Supplementary file 1 — Figs. S1 to S18 Table S1 [file sciadv.add2000_sm.pdf]

Science Advances
AAAS

Supplementary Materials for

Unlocking the in situ Li plating dynamics and evolution mediated by diverse metallic substrates in all-solid-state batteries

Can Cui *et al.*

Corresponding author: Tianyou Zhai, zhaity@hust.edu.cn; Huiqiao Li, hqli@hust.edu.cn

Sci. Adv. **8**, eadd2000 (2022)
DOI: 10.1126/sciadv.add2000

The PDF file includes:

Figs. S1 to S18
Table S1
Legends for movies S1 to S4

Other Supplementary Material for this manuscript includes the following:

Movies S1 to S4

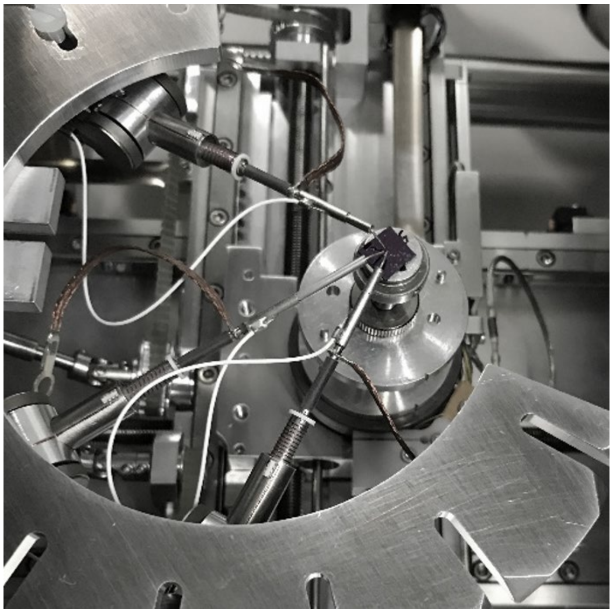

Fig. S1. Optical image of in situ SEM manipulation platform.

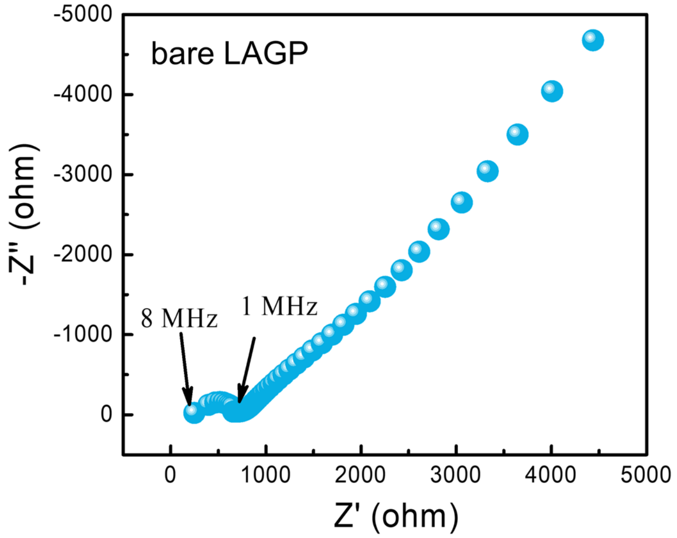

Fig. S2. EIS measurements of Au||LAGP||Au cell. The Li^+ conductivity of LAGP SSE is 3.10 × 10^{-4} S cm^{-1} at room temperature.

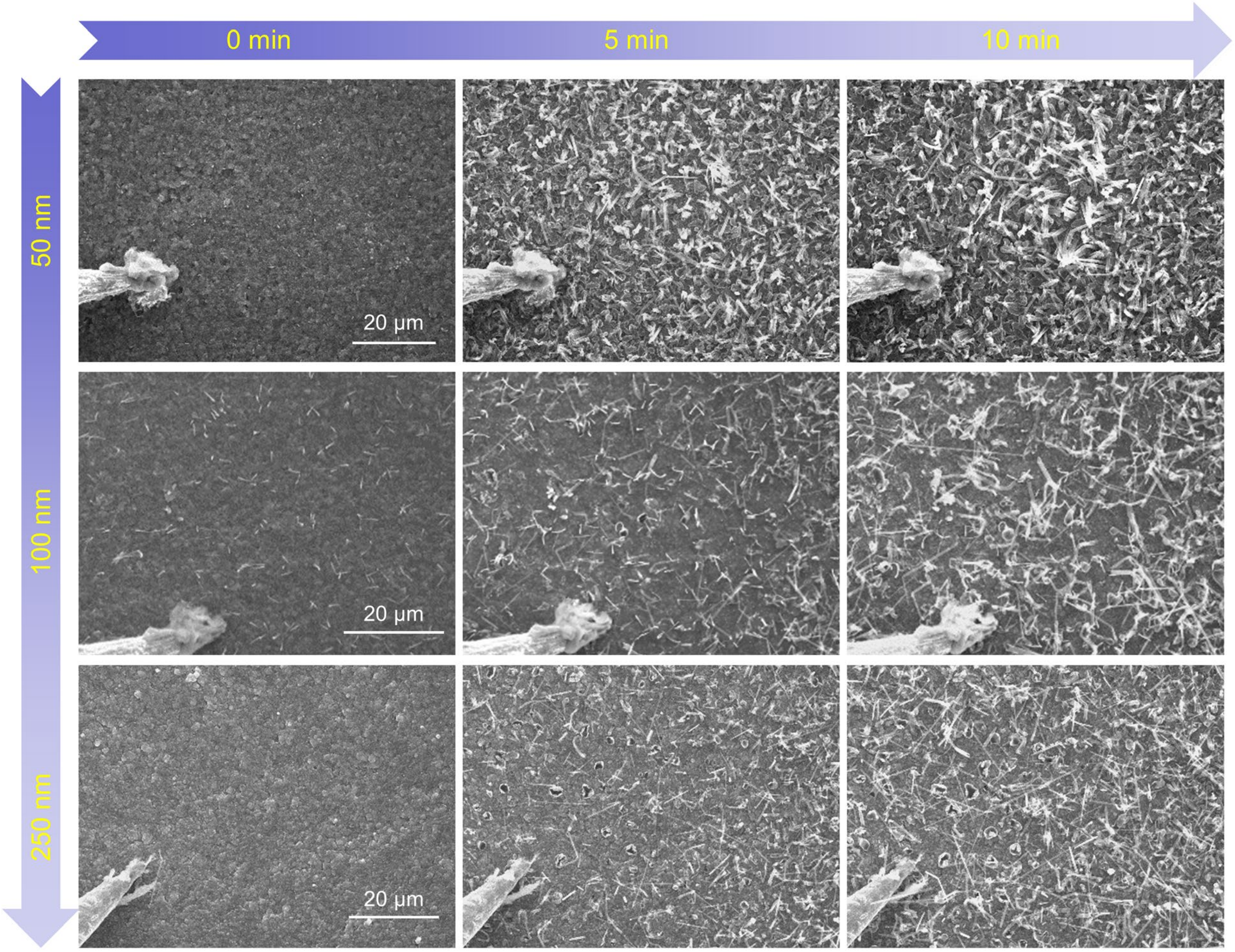

Fig. S3. Li plating behaviors on Cu substrate with a thickness of 50, 100, and 250 nm, respectively.

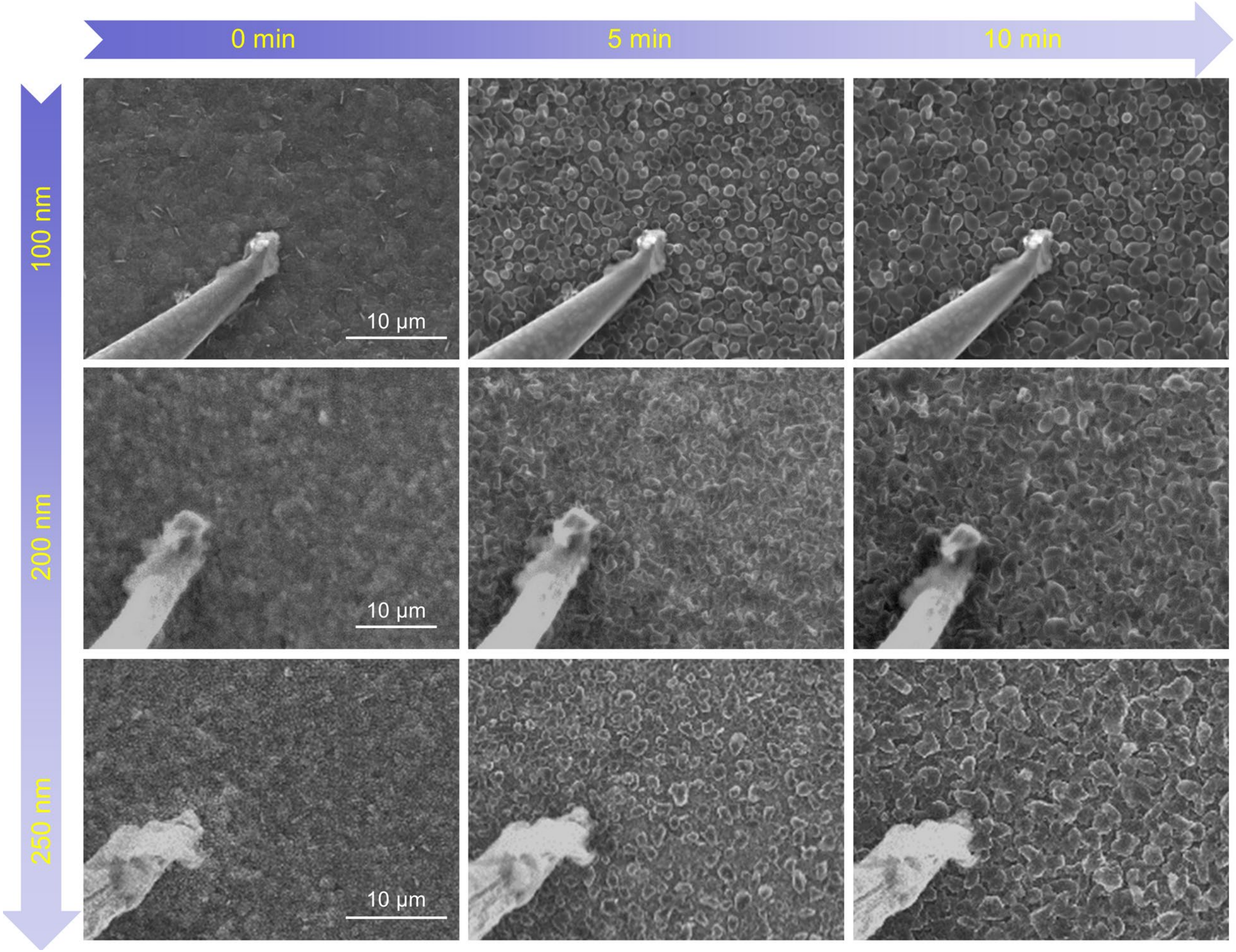

Fig. S4. Li plating behaviors on In substrate with a thickness of 100, 200, and 250 nm, respectively.

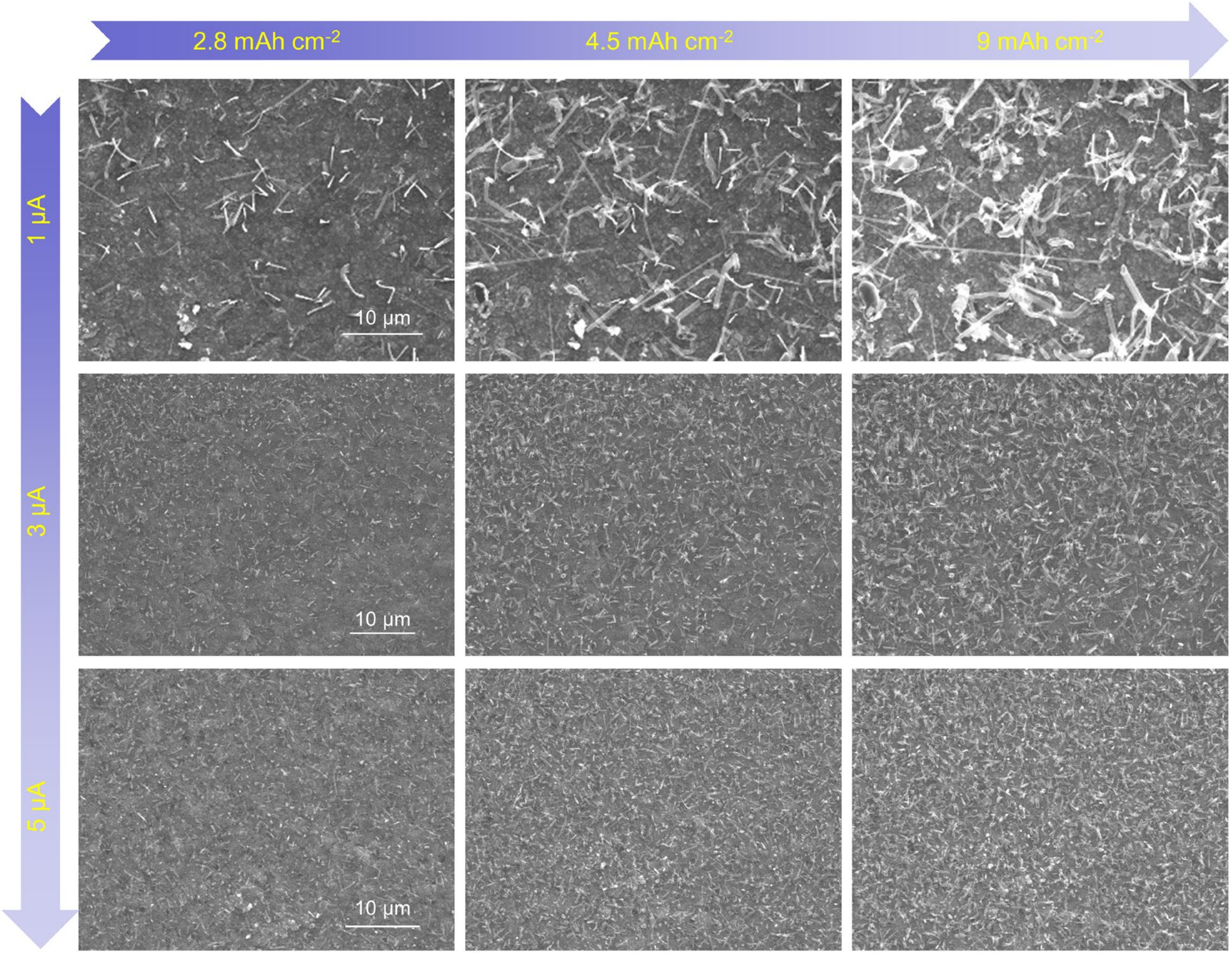

Fig. S5. The evolution of Li plating morphologies on Cu substrate under different currents, where SEM images show the Li deposition morphologies under constant capacity.

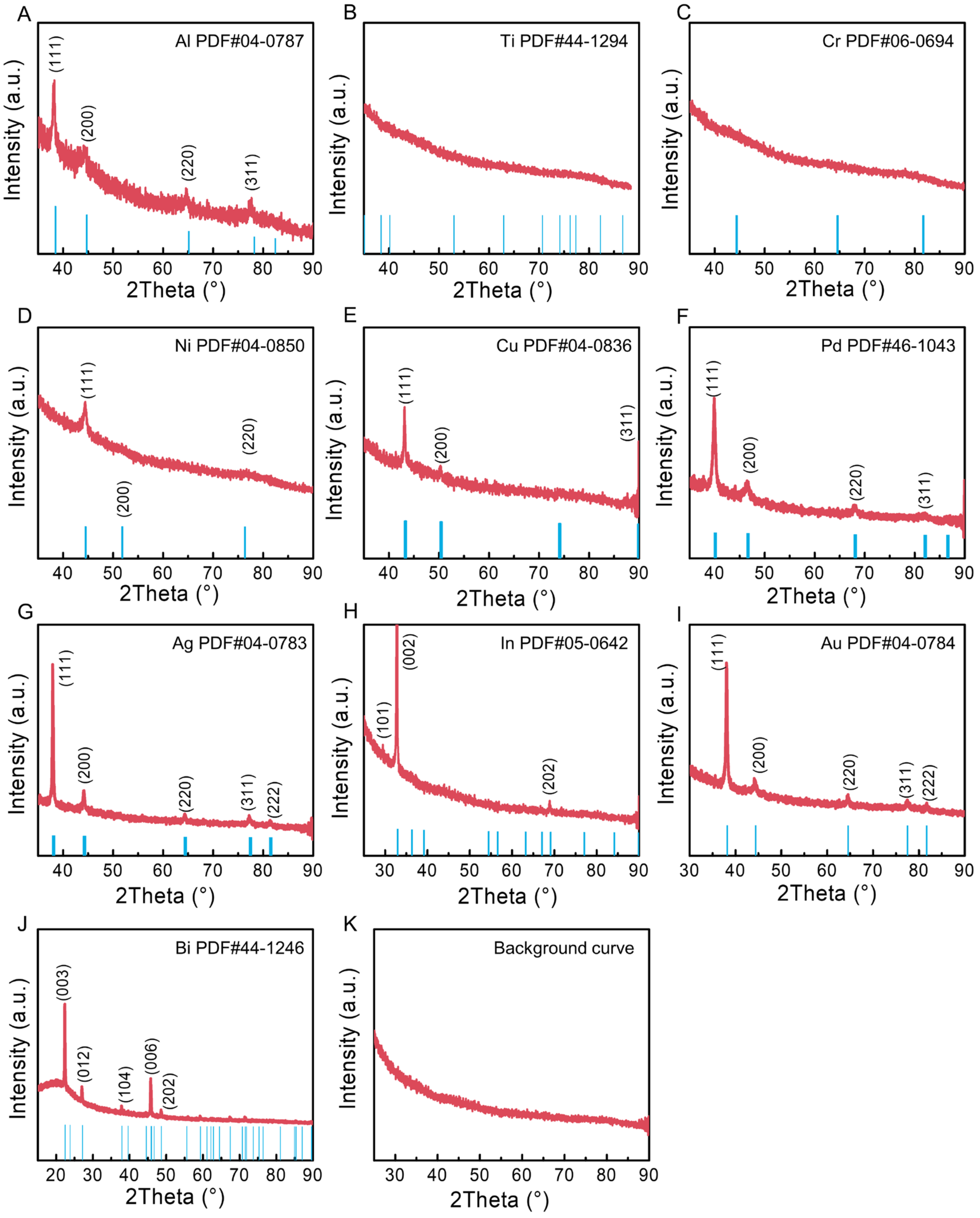

Fig. S6. XRD characterizations on 10 metallic substrates. XRD patterns of Al (**A**), Ti (**B**), Cr (**C**), Ni (**D**), Cu (**E**), Pd (**F**), Ag (**G**), In (**H**), Au (**I**), and Bi (**J**) electron-beam evaporated metallic films. (**K**) Background curve.

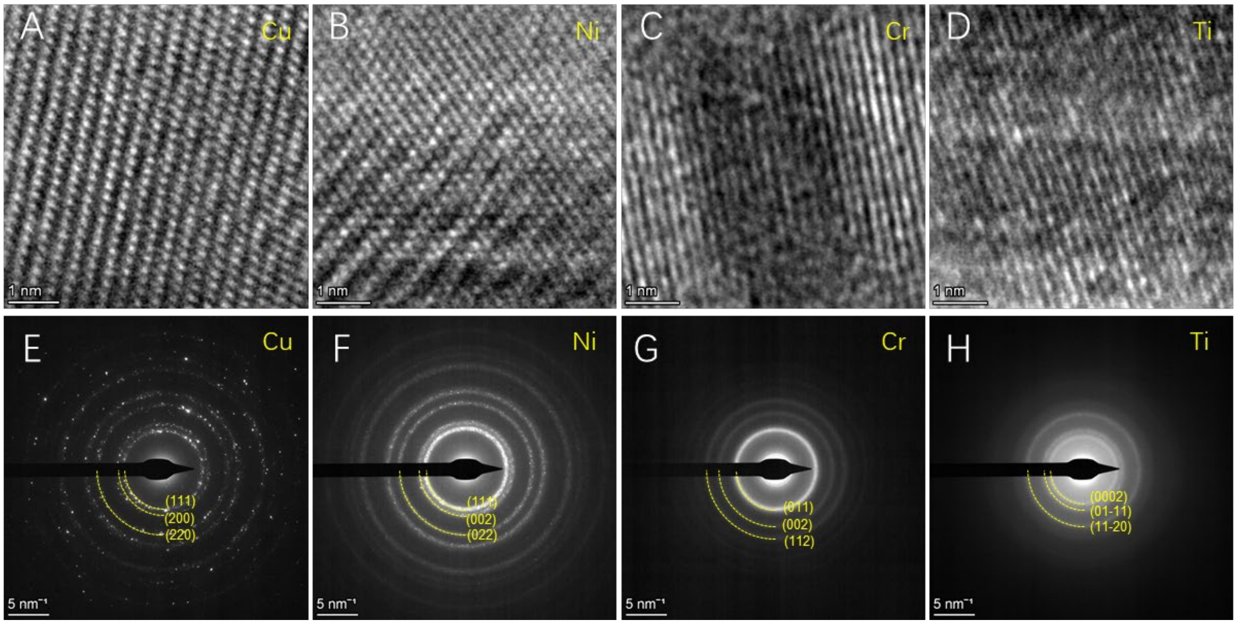

Fig. S7. HRTEM and SAED of some evaporated metallic substrates. HRTEM images of evaporated Cu (**A**), Ni (**B**), Cr (**C**), and Ti (**D**) metallic substrates. And the corresponding SAED patterns of Cu (**E**), Ni (**F**), Cr (**G**), and Ti (**H**) substrates.

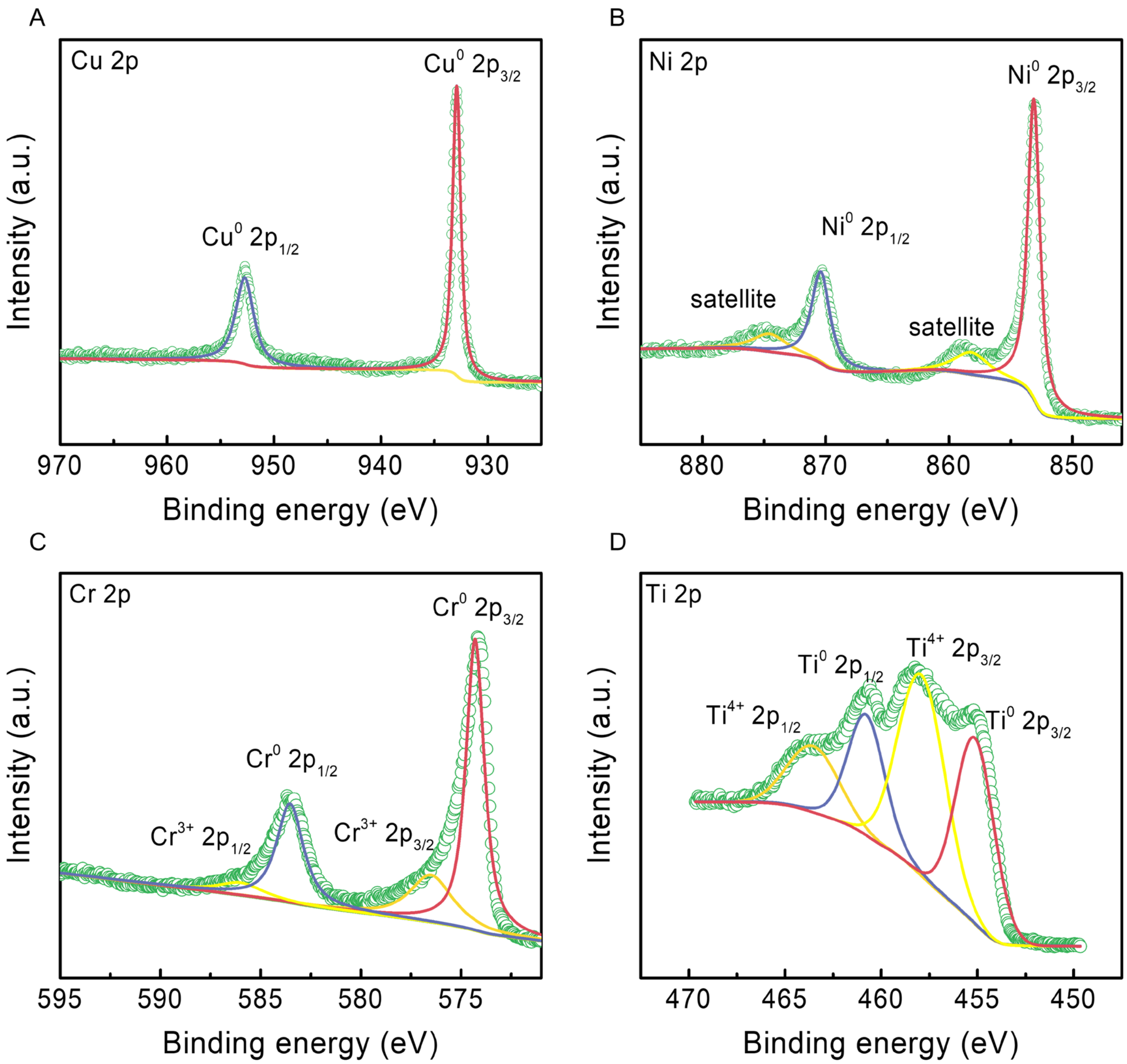

Fig. S8. XPS spectrums of Cu 2p (A), Ni 2p (B), Cr 2p (C), and Ti 2p (D).

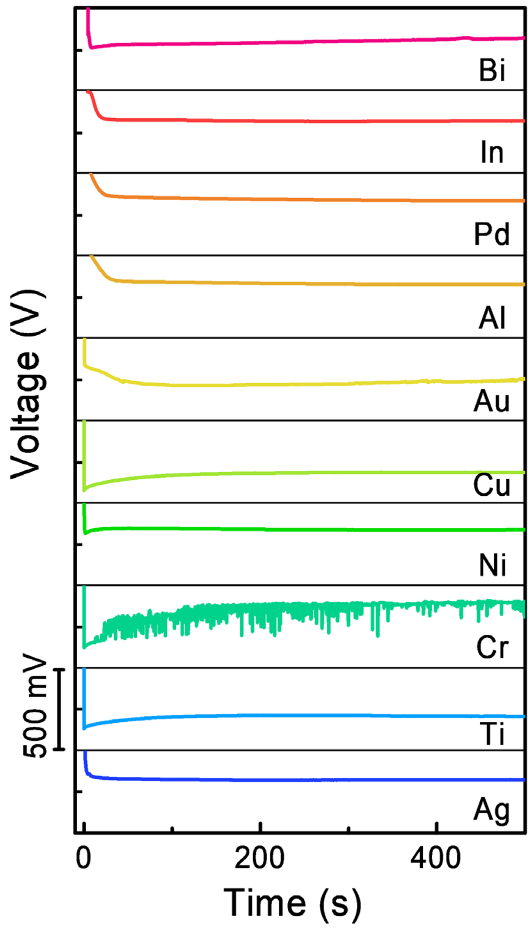

Fig. S9. Discharging curves of various metals with Li, where the scales in all discharging curves are from -500 to 0 mV.

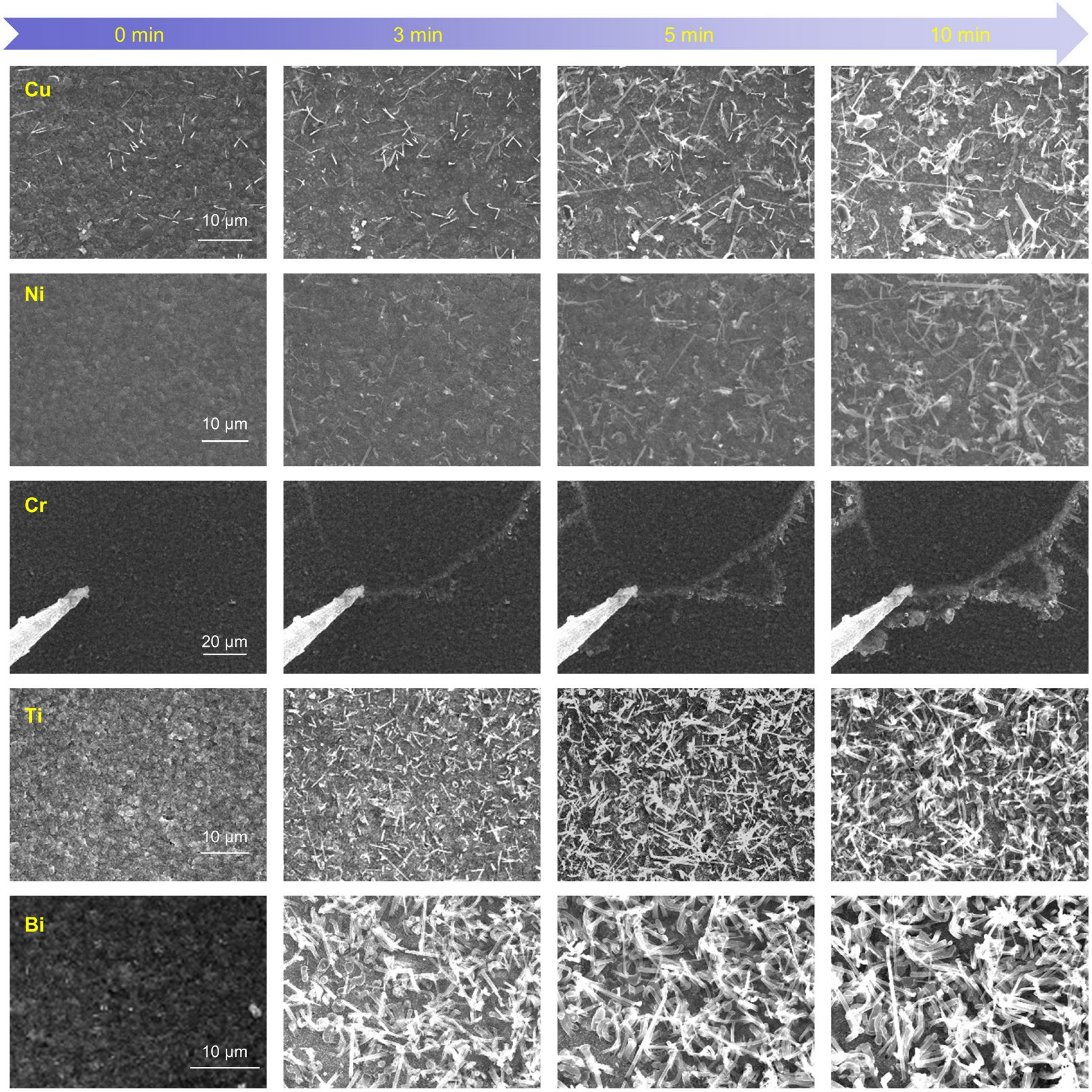

Fig. S10. The dynamic process of Li nucleation and growth on Cu, Ni, Cr, Ti, and Bi metallic substrates for 0, 3, 5, and 10 min, respectively.

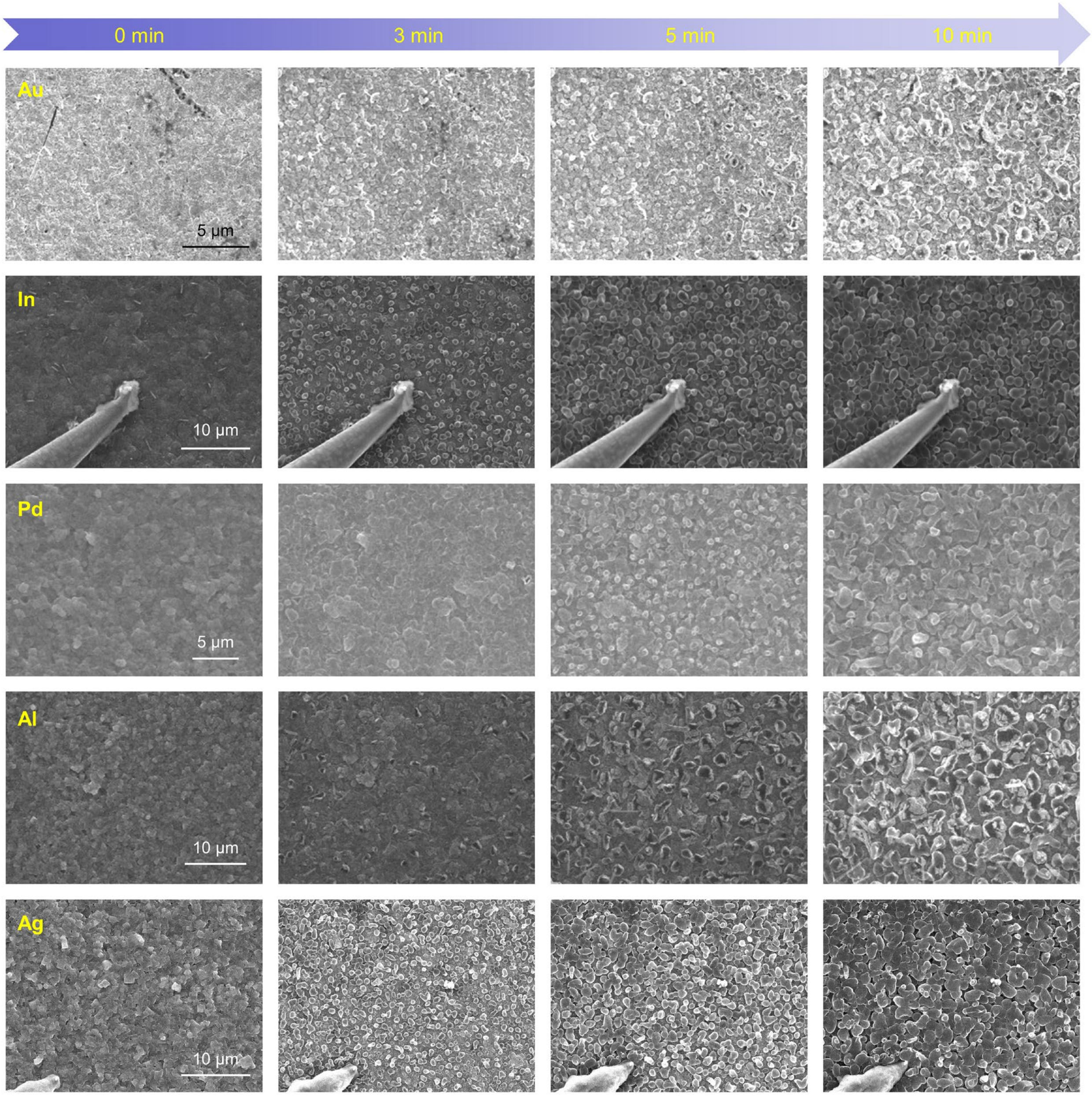

Fig. S11. The dynamic process of Li nucleation and growth on Au, In, Pd, Al, and Ag metallic substrates for 0, 3, 5, and 10 min, respectively.

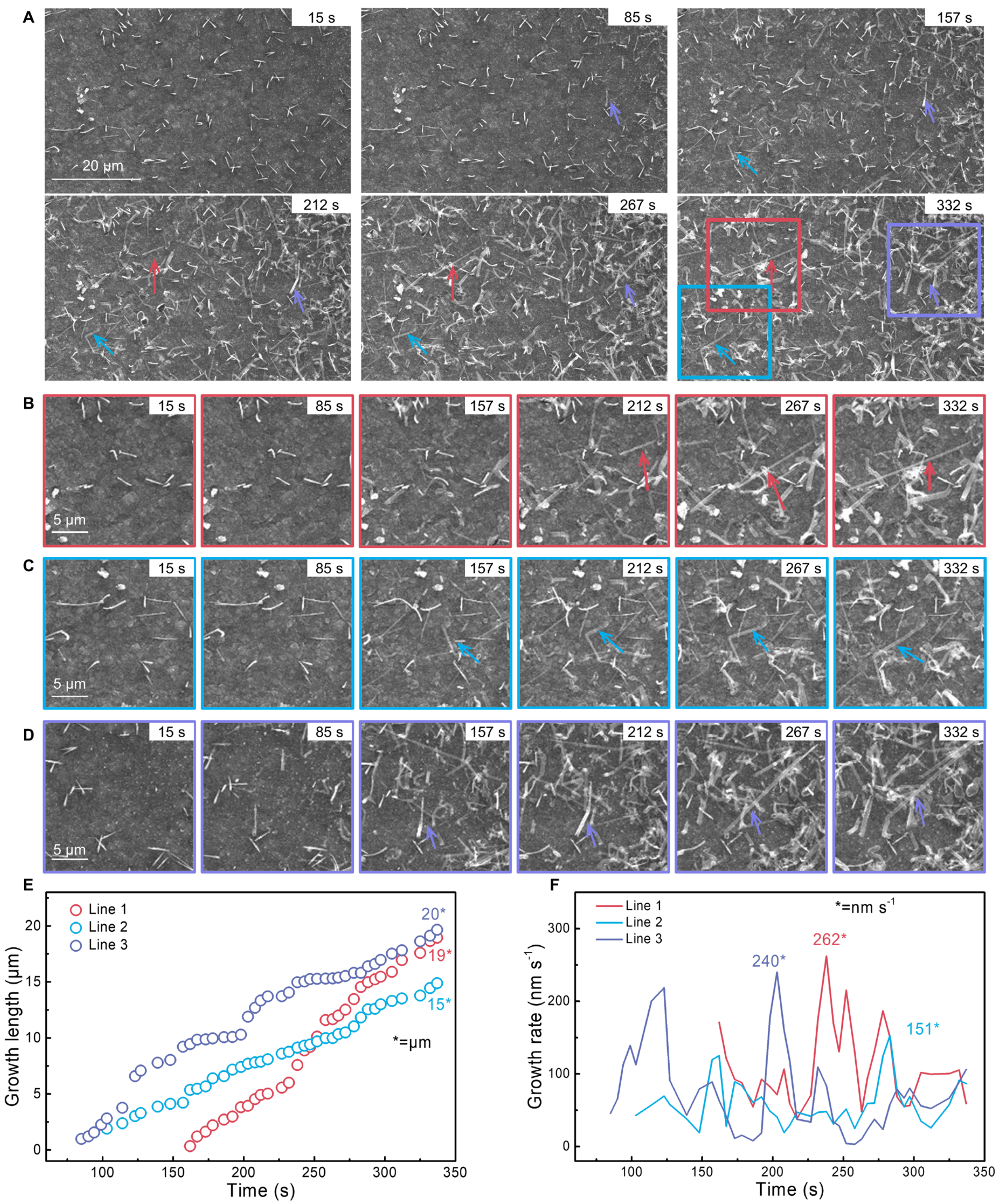

Fig. S12. Li growth behavior on Cu substrate. (**A**) Time-series SEM images of Li dendrites on Cu substrate. The randomly chosen areas of three individual Li whiskers are enlarged in red (**B**), blue (**C**), and purple (**D**) square frames, respectively. (**E**) The evolution of the measured growth lengths of Li whiskers along time. (**F**) The growth rates of Li whiskers versus time. To be noted, Line 1, 2, 3 correspond to SEM images of red (**B**), blue (**C**), and purple (**D**), respectively.

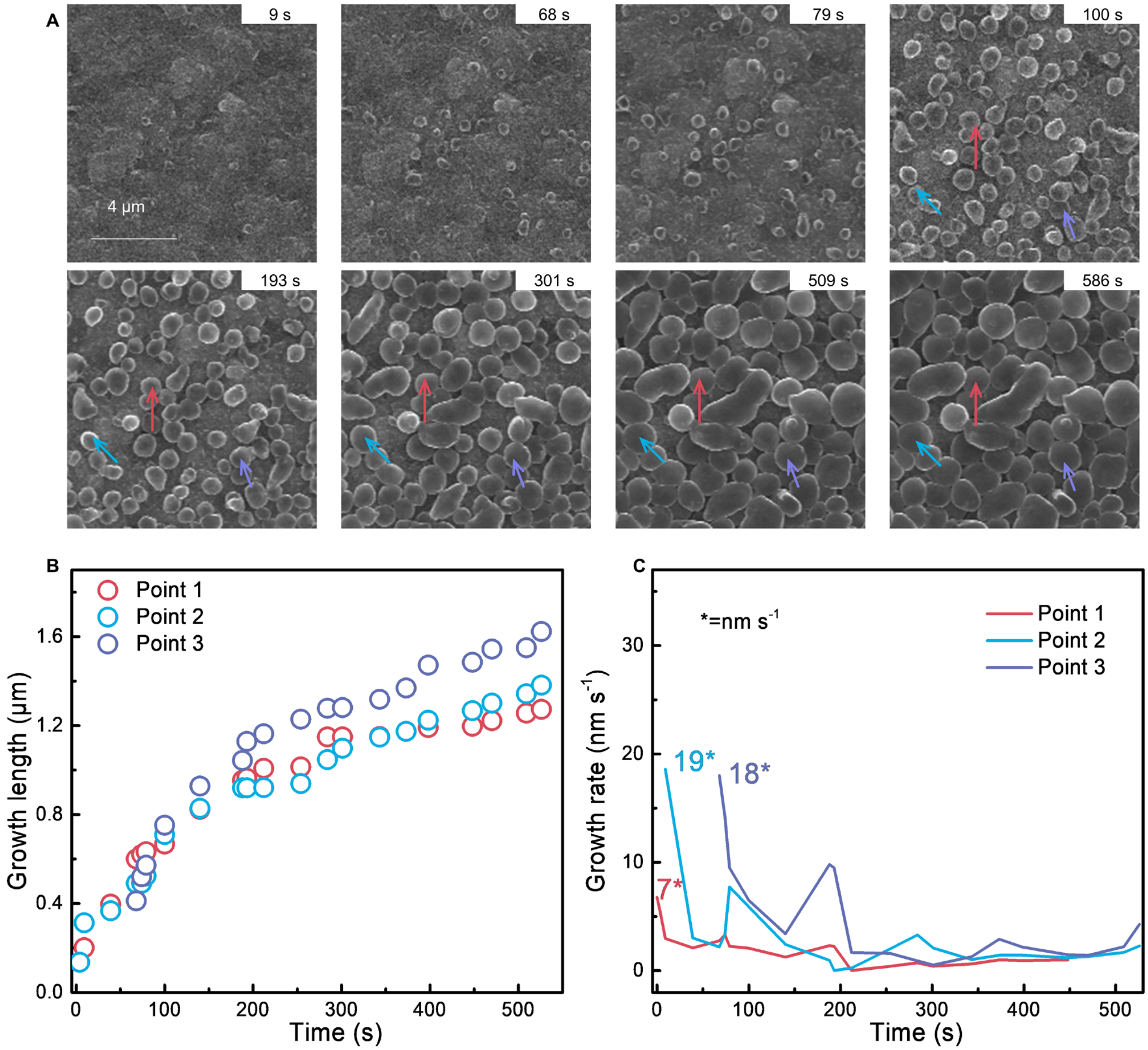

Fig. S13. Li growth behavior on In substrate. (**A**) Time-series SEM images of in situ growth of Li particles on In substrate. The selected particles are pointed by arrow. (**B**) The evolution of growth lengths of the randomly chosen Li particles versus time. (**C**) The growth rate of the chosen Li particles versus time. To be noted, Point 1, 2, 3 correspond to the particles pointed by red, blue, and purple arrows in (**A**), respectively.

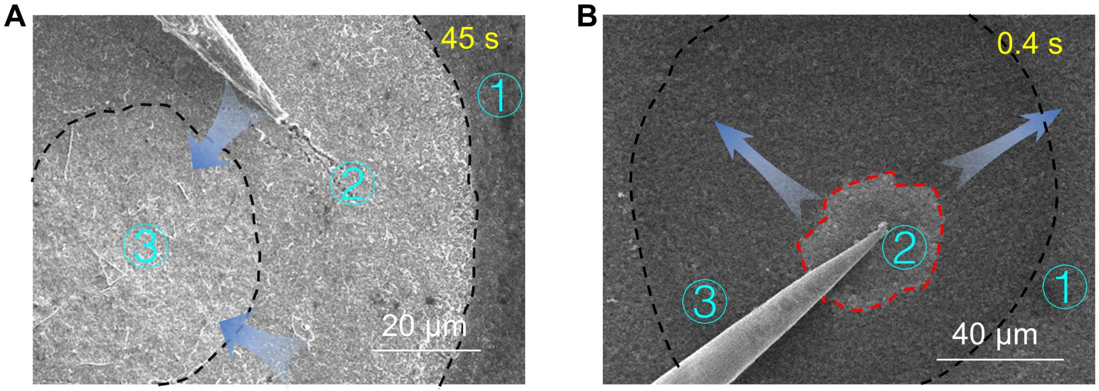

Fig. S14. SEM images of Li-Au and Li-In alloying. (**A**) Li-Au alloying at 45 s. (**B**) Li-In alloying at 0.4 s. For clarity, area ① is the naked LAGP SSE surface; area ③ is the deposited metallic electrode on LAGP SSE surface, namely non-lithiated area; area ② is the reacted area, where metallic electrode (M) is lithiated to Li-M alloy.

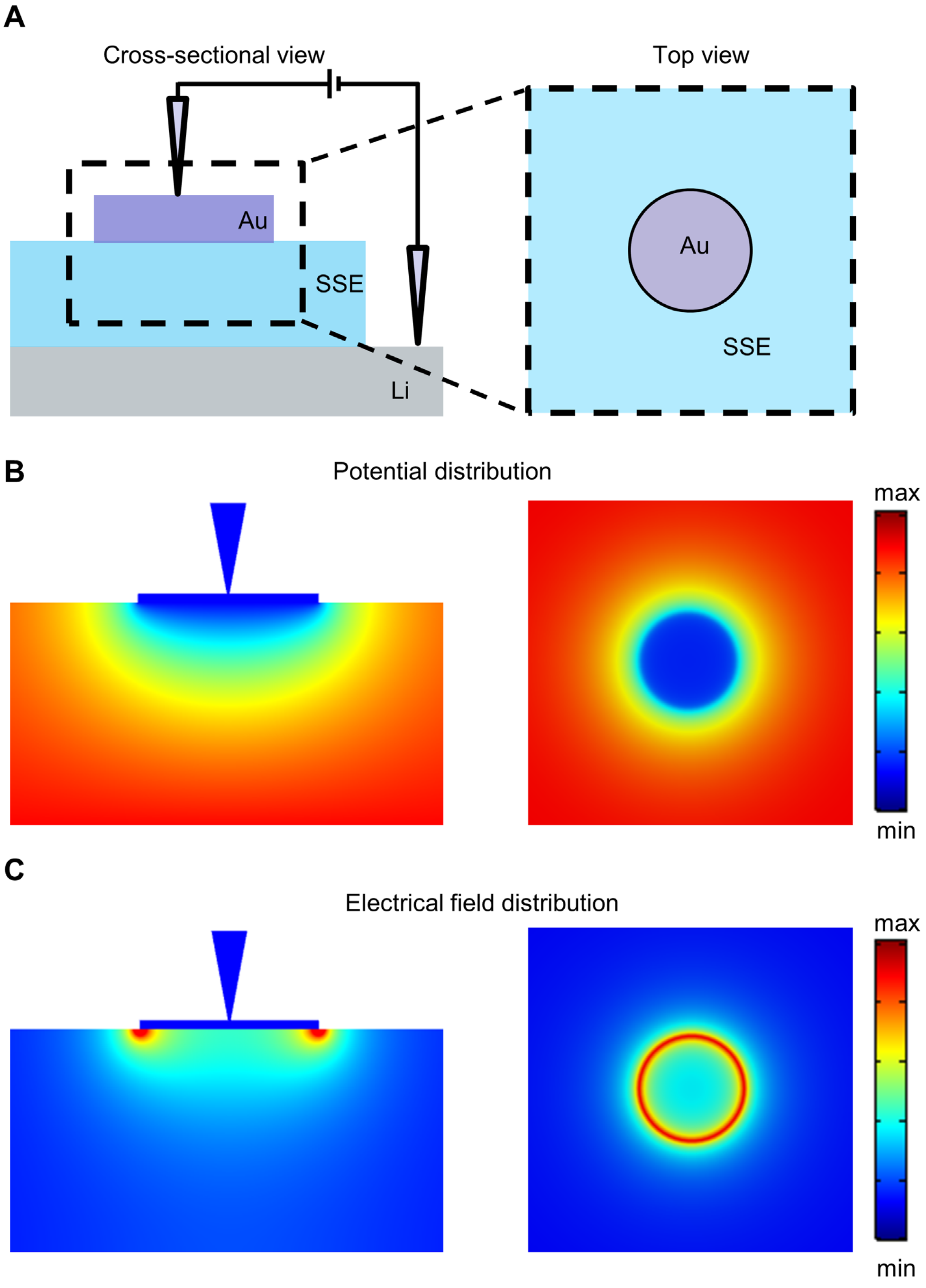

Fig. S15. The potential and electrical field distributions in SSE with Au electrode. (**A**) Schematic diagram of Au||SSE||Li simulated model. (**B**) The potential distribution in the SSE beneath the Au electrode. (**C**) The electrical field distribution in the SSE beneath the Au electrode.

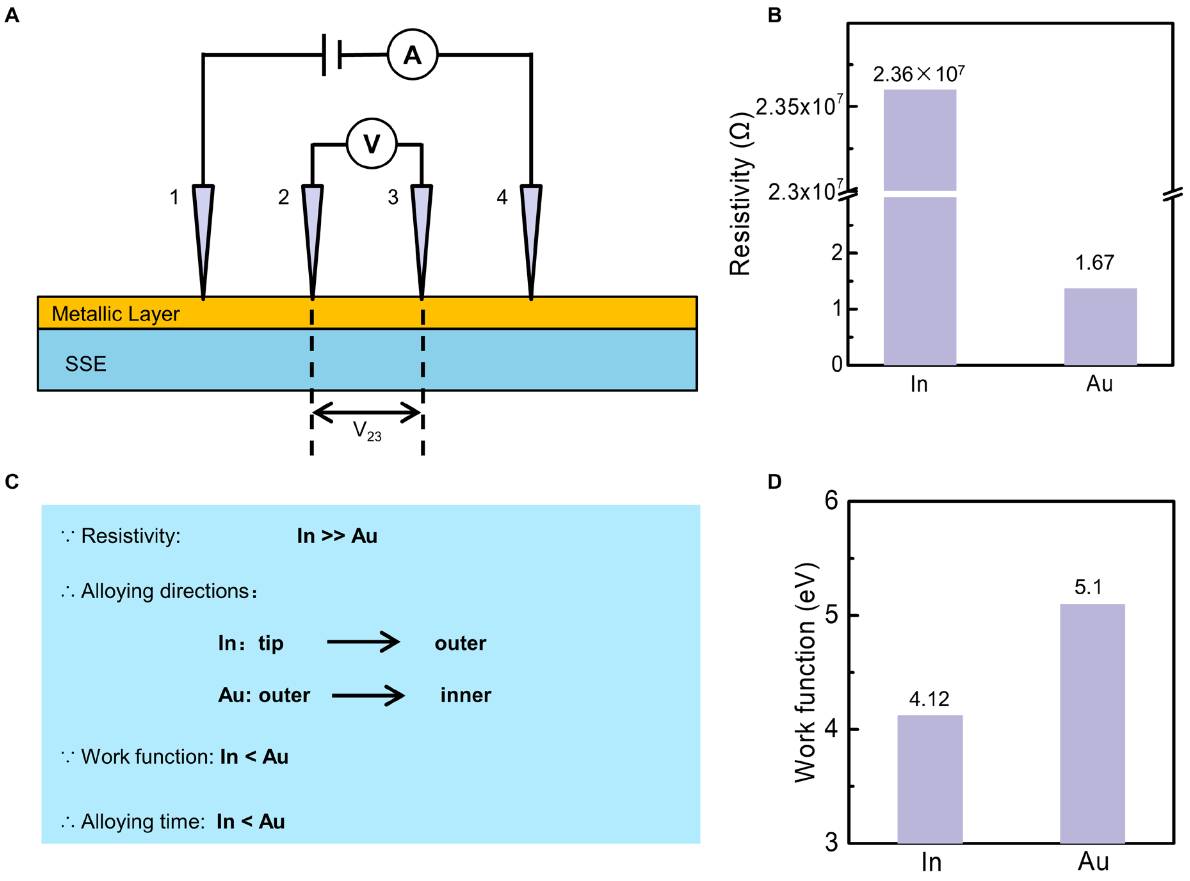

Fig. S16. Comparison of In and Au metallic layer in resistivity and work function. (**A**) Schematic diagram of four-point probe method. (**B**) Resistivities of In and Au metallic layer. (**C**) Summary of the alloying phenomena and reasons. (**D**) Work functions of In and Au metals.

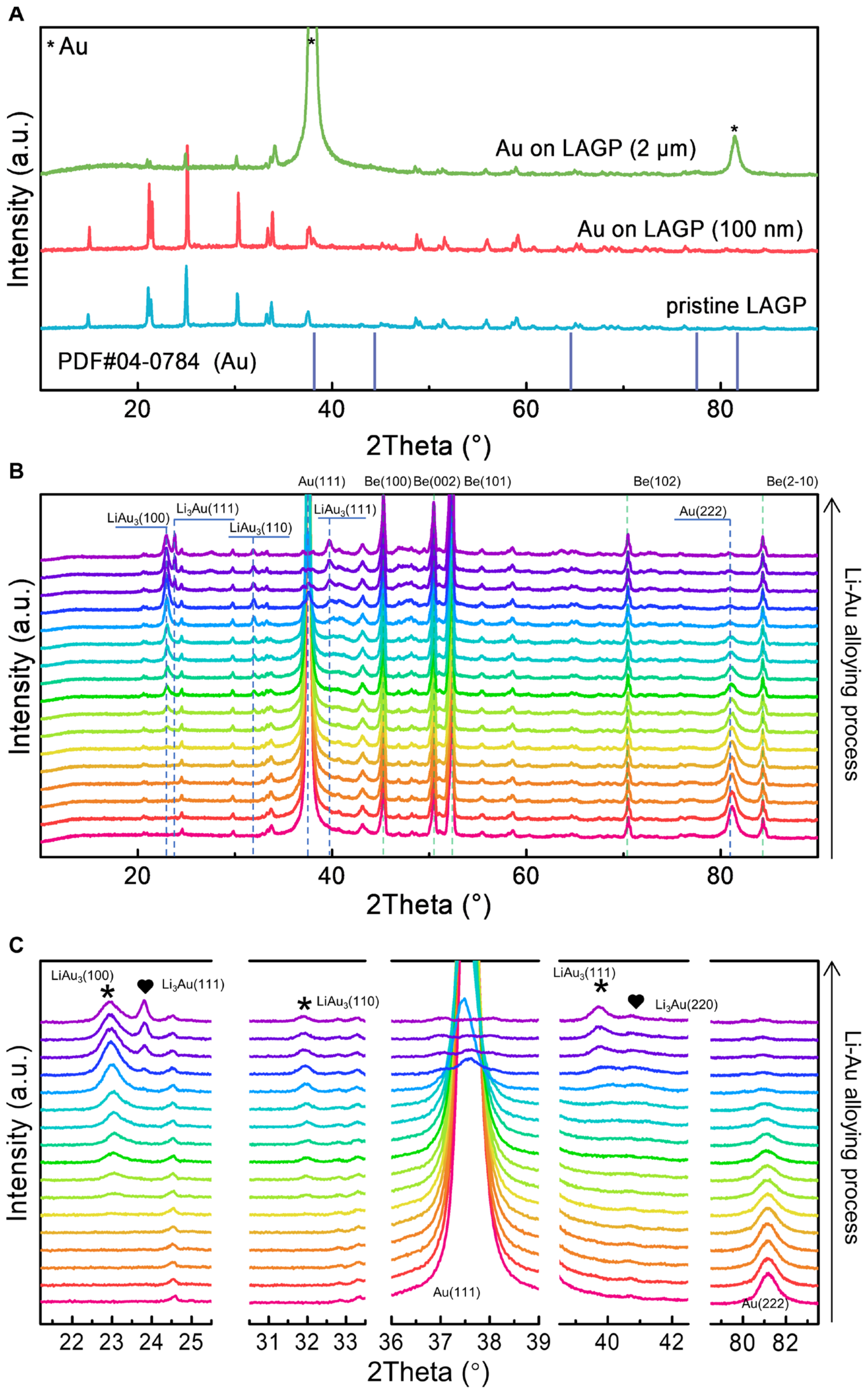

Fig. S17. In situ XRD characterizations for Li-Au alloying process. (**A**) XRD patterns of Au with different thicknesses on LAGP. The substrate thickness used in XRD characterizations is 2 μm for better detection of the corresponding diffraction peaks. (**B**) In situ XRD spectra of Li-Au alloying process. And (**C**) is the enlarged spectra.

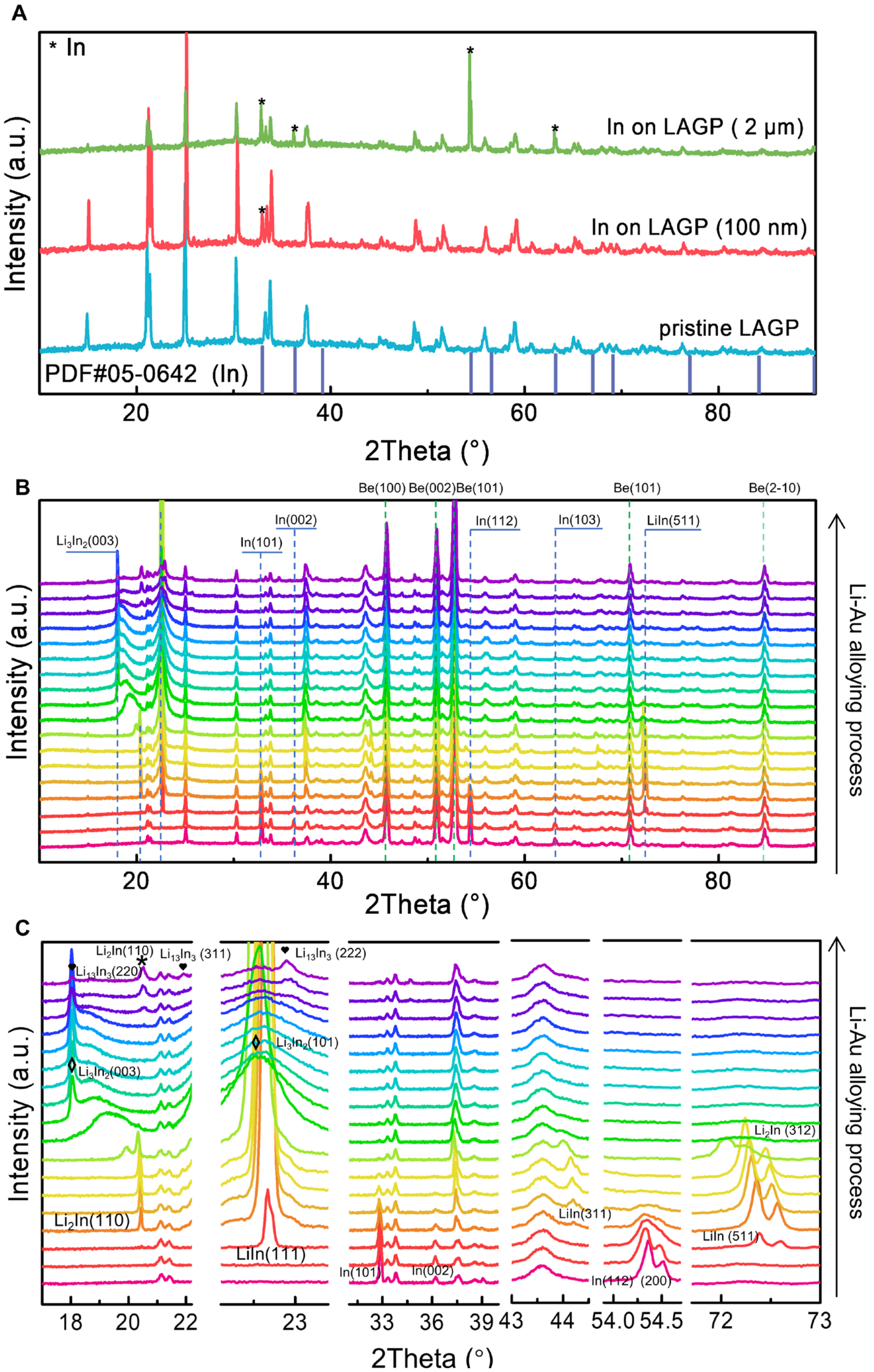

Fig. S18. In situ XRD characterizations for Li-In alloying process. (**A**) XRD patterns of In with different thicknesses on LAGP. The substrate thickness used in XRD characterizations is 2 μm for better detection of the corresponding diffraction peaks. (**B**) In situ XRD spectra of Li-In alloying process. And (**C**) is the enlarged spectra.

Table S1.
The fundamental physical properties and key parameters of the metals.

Number	Element	r (pm)	a (Å)	b (Å)	c (Å)	Crystal structure	PDF-cif
1	Li	152	3.51	3.51	3.51	bcc	15-0401
2	Bi	154.7	4.55	4.55	11.86	hcp	44-1246
3	In	167	3.25	3.25	4.95	bcc	05-0642
4	Ag	144	4.09	4.09	4.09	fcc	04-0783
5	Pd	137	3.89	3.89	3.89	fcc	46-1043
6	Al	143.1	4.05	4.05	4.05	fcc	04-0787
7	Au	144	4.08	4.08	4.08	fcc	04-0784
8	Cu	128	3.62	3.62	3.62	fcc	04-0836
9	Ni	124	3.52	3.52	3.52	fcc	04-0850
10	Cr	128	2.88	2.88	2.88	bcc	06-0694
11	Ti	147	2.95	2.95	4.68	hcp	44-1294

Movie S1. The dynamic heterogeneous dendrite-like Li growth mode. Galvanostatic current of 1 μA is applied on the Li||SSE||Bi all-solid-state battery device with Li as the counter/reference electrode and Bi substrate as the working electrode. An in situ SEM video shows typical dendrite-like Li growth. The Li dendrites grow vertically to the substrate. The video was recorded at 10 frames per second and played at 35 × speed.

Movie S2. The dynamic homogeneous and uniform particulate-like Li growth mode. Galvanostatic current of 1 μA is applied on the Li||SSE||In all-solid-state battery device with Li as the counter/reference electrode and In substrate as the working electrode. An in situ SEM video shows typical uniform particulate-like Li growth. The Li particles grow laterally along the substrate. The video was recorded at 10 frames per second and played at 35 × speed.

Movie S3. The dynamic Li-Au alloying process. An in situ SEM video shows the dynamic alloying process of Li-Au at the initial discharging process before Li nucleation and growth. The video was recorded at 10 frames per second and played at 8 × speed.

Movie S4. The dynamic Li-In alloying process. An in situ SEM video shows the dynamic alloying process of Li-In at the initial discharging process. The alloying process can be tracked by the fast and dynamic contrast variation of the substrate micro-electrode. The video was recorded at 10 frames per second and played at 1 × speed.
